# Continuous-Flow Photochemical Isomerization of Humulones to Isohumulones

**DOI:** 10.3390/molecules30051002

**Published:** 2025-02-21

**Authors:** Bruce C. Hamper, Bradley Gallow, Gregory Giovine, Trevor Smith

**Affiliations:** Department of Chemistry & Biochemistry, University of Missouri-St. Louis, 43 Benton Ct., St. Louis, MO 63121, USA

**Keywords:** humulone, isohumulone, photochemistry, flow chemistry, green chemistry, photoisomerization, light emitting diodes (LED)

## Abstract

Humulones are a family of homolog natural products obtained from the strobiles of humulus lupulus, or hops plants. Structurally, they consist of substituted phloroglucinols with two isoprenyl side chains, a carbonyl group and a quaternary ring carbon substituted with a hydroxyl group. The three most prominent homologs are n-, co- and ad-humulone, containing isobutyl, isopropyl and secbutyl ketone groups, respectively. When solutions of humulones are exposed to UV light, they undergo stereoselective isomerization to the five-membered ring trans-isohumulones. A photoreactor was assembled from strip LEDs in close contact with UV-transparent tubing. This reactor allowed continuous-flow chemical synthesis of the isohumulones. The yield, conversion and product throughput are compared for the humulones, using LEDs emitting white, blue and ultraviolet light (visible, 400 nm, and 365 nm, respectively). Using an optimized continuous-flow reactor, a throughput of 0.43 g/h was obtained for trans-n-isohumulone.

## 1. Introduction

**Figure 1:**
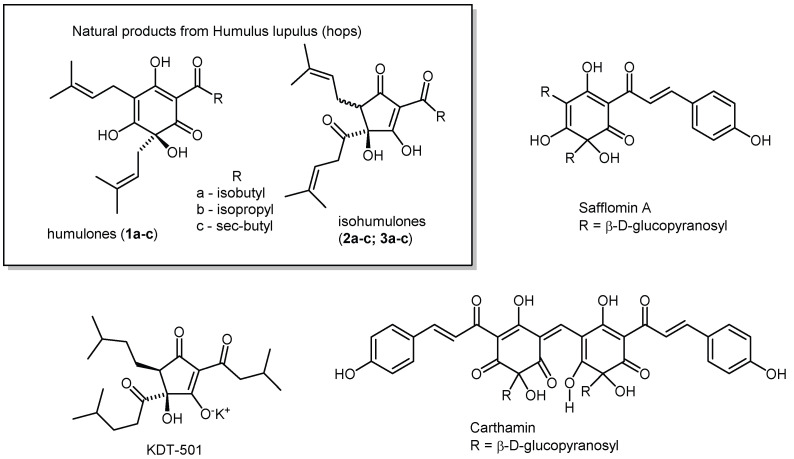
Structure of selected oxidized phloroglucinols and cyclopentenone products.

Substituted oxidized phloroglucinols have been identified in a number of plant sources, including humulones from hops (humulus lupulus), and safflomin A and carthamin from safflower (carthamustinctorius) [1,2,3,4,5,6]. (Figure 1) The harvested strobiles of hop plants contain from 2 to 15% humulones by dry weight [7]. Hops are one of four essential ingredients in beer brewing, along with malted grains, water and yeast. The humulones in hops are extracted into wort during beer brewing and isomerized to isohumulones [8,9,10,11]. It is the isohumulones that provide bitter flavor, as the humulones themselves have negligible bitterness, based on sensory perception [12,13]. By contrast, the products from safflower are used primarily as dyes. Safflomin A is a yellow pigment from the flowers of safflower. Carthamin has been used as a red dye in the textile industry and is also a food coloring agent known as Natural Red 26 [14,15]. Isomerization of oxidized phloroglucinols to five-member ring cyclopentenones is well known in the case of humulones to isohumulones [16] and has been observed in the synthesis of the derivatives safflomin and carthamin [17,18]. Investigations of these natural products and their derivatives are of great interest, due to the potential for biological activity [19]. For instance, isohumulones not only give bitter flavor, but also provide antibacterial protection in beer, improving long-term storage ability and product stability [20,21,22]. Isohumulones have also been shown to have potential for control of non-alcoholic fatty acid liver disease (NAFLD), type 2 diabetes and Alzheimer’s disease [23,24,25]. Hydrogenation of cis-isohumulone provided tetrahydro-cis-isohumulone KDT 501 (Kindex Therapeutics), which was evaluated in phase II clinical trials for treatment of insulin resistant, prediabetic patients, and is also being considered for control of polycystic ovarian syndrome and NAFLD [26,27,28,29].

Since the seminal report from Booker-Milburn in 2005, Ref. [30], photoreactions coupled with flow chemistry have emerged as facile methods usable for the preparation of a wide range of compounds [31,32,33,34,35]. Light sources can be regarded as renewable, traceless reagents; as a consequence, most photochemical processes adhere to fundamental principles of “green chemistry”. Unless sunlight is being used, however, the light sources require energy input. Therefore, it is important to use the most efficient source possible and to minimize the distance between the light source and the substrate molecules. Most recent photochemical publications have employed reactions with photocatalysts [36]. However, it is also possible to facilitate molecular rearrangements and isomerization for molecules that absorb light in the appropriate region on the spectrum [37,38,39,40].

**Scheme 1:**
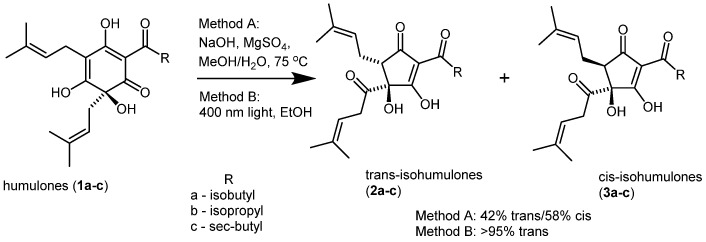
Isomerization of humulones to isohumulones; thermal and photochemical reactions.

**Scheme 2:**
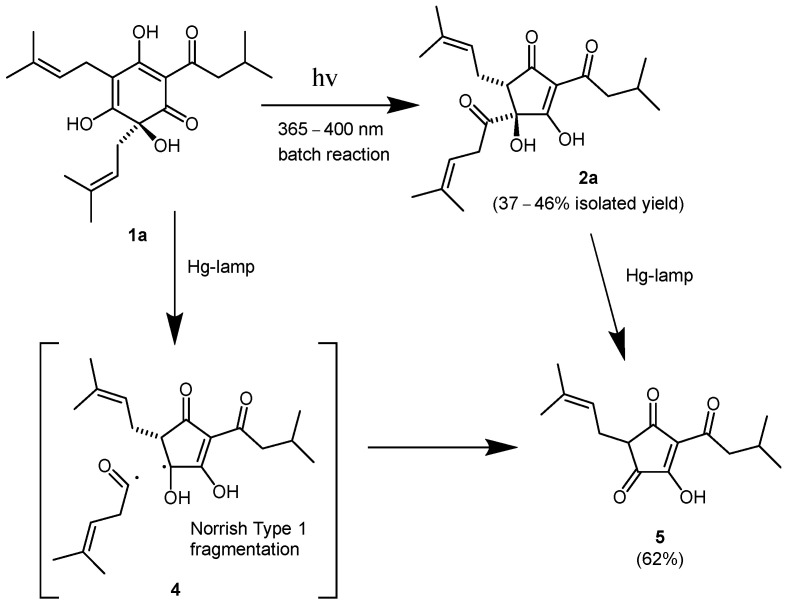
Norrish type 1 fragmentation of isohumulone intermediate.

Humulones (**1**) can be isomerized to isohumulones (**2** and **3**) by both thermal and photochemical methods (Scheme 1) [41]. Thermal isomerization typically provides a mixture of trans (**2**) and cis (**3**) isomers, with the ratio dependent on the use of catalysts and reaction conditions [42]. In 1965, Clarke and Hildebrand reported the stereoselective photochemical isomerization of **1a** to the trans isomer **2a** in 46% isolated yield using an incandescent lamp [43]. Sharpe and Ormrod also reported photochemical preparation of **2a** from *n*-humulone **1a** using a filtered, medium pressure Hg lamp; however, isolated yields were also modest, at 37% (Scheme 2) [44]. As a consequence of the low yields from photochemical methods, preparation of trans-isohumulones as standards for the food and beverage industry have relied on the thermal isomerization followed by selective fractional crystallization of the trans isomers [45]. In this report, we present the continuous-flow photochemical approach for preparation of trans-isohumulones in a scalable, reproducible and high-yield manner.

## 2. Results and Discussion

For our initial investigation of the photochemistry of humulone (**1a**), we used an unfiltered, water-cooled medium pressure Hg lamp in a batch reactor. A 0.17 M solution of **1a** in methanol was exposed to the Hg lamp for 24 h, resulting in complete conversion to a new product. Chromatographic purification resulted in a moderate yield of cyclopentenone (**5**) (Scheme 2). HPLC analysis of the crude reaction mixture did not show any of the desired isohumulone (**2a**). Due to the wide spectrum emission of the mercury lamp, it is likely that the isohumulone (**2a**) is an intermediate which undergoes further reaction to afford the dehydrohumulinic acid (**5**). A Norrish type 1 fragmentation (**4**) gives rise to two free radicals via homolytic cleavage and loss of the hexenone side chain. Further loss of a hydrogen atom gives the observed dehydrohumulinic acid (**5**). The Norrish type 1 fragmentation is well known and is responsible for the undesirable light-struck flavors in beer [46,47].

In order to obtain more precise control of the light source emission, we investigated custom-built reactors using commercially available LEDs. Our first batch reactor was constructed using a short length of a strip of UVA400 nm LEDs wrapped around a 40 mL sample vial [9]. This was effective for carrying out isomerization of *n*-humulone (**1a**) to *n*-isohumulone (**2a**) on a small scale and was particularly useful for reactions run in deuterated solvents where larger amounts of solvent might be cost prohibitive. However, attempts at larger scale reactions in the batch reactor led to complex mixtures and lower yields. Flow reactors have an advantage in such situations by providing greater control of the exposure time and improvements in the distance of the photon source to the reaction mixture. LEDs, in particular, have advantages over other light sources, providing narrow spectral output (±10 nm for UVA sources), efficient conversion of electrical energy to light and relatively low levels of heat generation.

**Figure 2:**
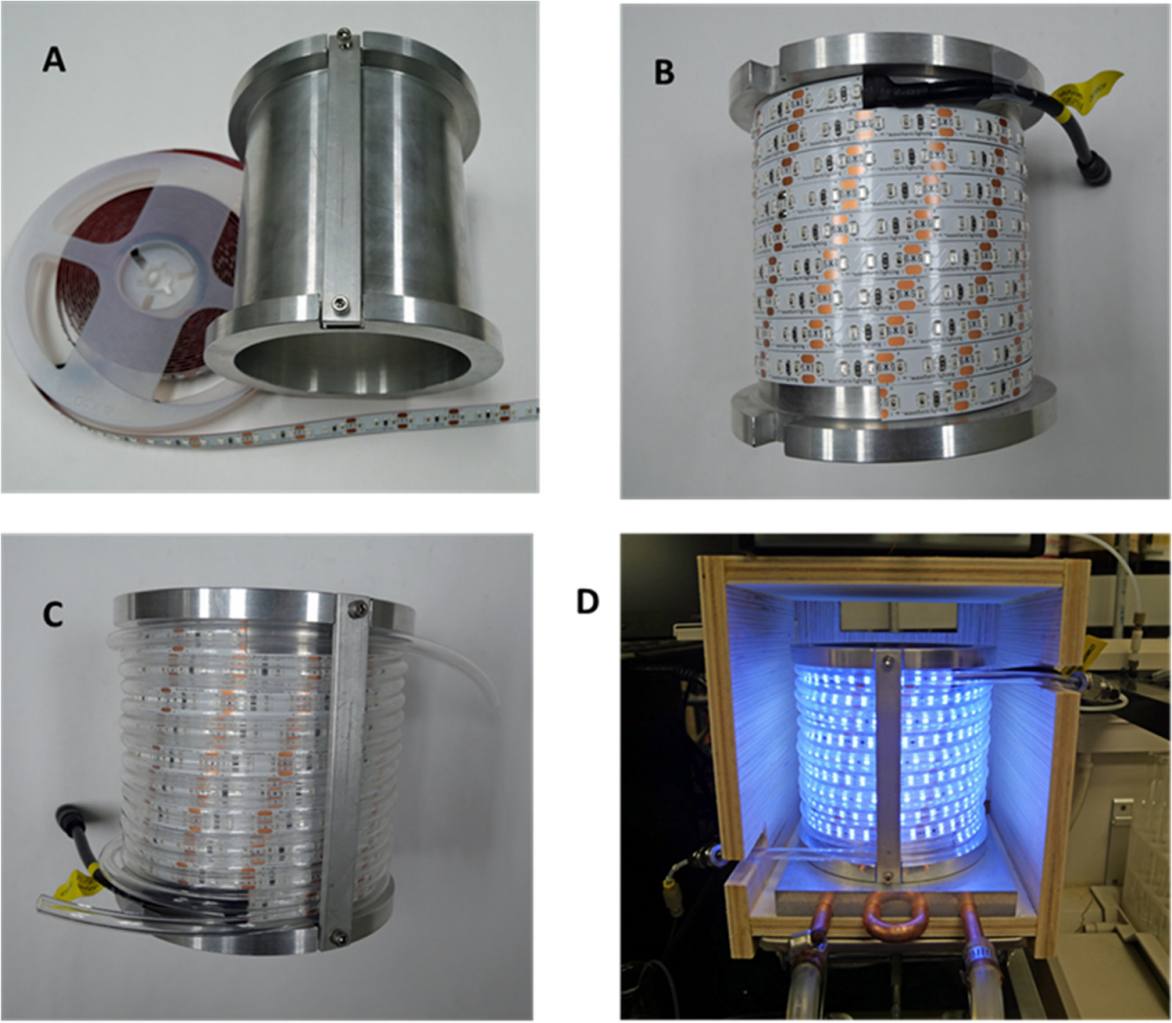
Construction of the continuous-flow photoreactors: (**A**) aluminum support; (**B**) strip LEDs attached to aluminum cylinder; (**C**) UV-transparent tubing held in place by aluminum bar; and (**D**) completed photoreactor with aluminum/copper cooling plate and safety cabinet (door open for photo).

**Figure 3:**
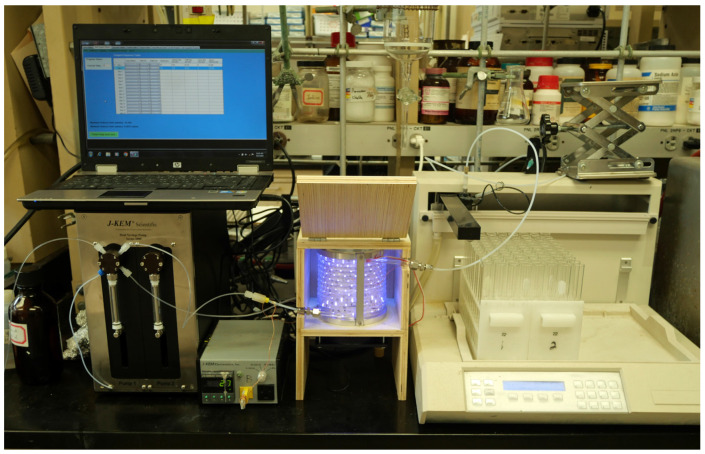
Continuous photoflow system, including programmable dual syringe pumps, temperature-controlled LEDs, UV-transparent THV tubing and fraction collector.

Continuous-flow photoreactors using LEDs have been reported for numerous photoflow reactions [35], including [2 + 2] cycloadditions [38,48], preparation of APIs [49,50] and transformations using photocatalysts [51,52]. For our studies, we built four flow photoreactors (A, B, C and D) using an aluminum base [53], strip LEDs and UV-transparent THV polymer tubing (Figure 2). Addition of samples to the flow reactor and collection of the products were provided by computer controlled, programmable syringe pumps and a fraction collector (Figure 3). Details are provided in the Supplementary Materials.

Isomerization of *n*-humulone (**1a**) to trans-*n*-isohumulone (**2a**) was investigated using four flow photoreactors for the determinations of starting material conversion and isolated product yield (Table 1). Each run was carried out by introduction of a solution of **1a** in ethanol and fractionated collection of the product over the course of the run. The relatively large internal diameter of the THV tubing resulted in significant diffusion of the sample, such that a 1.0 mL injection resulted in the collection of the product in 50 mL of reactor eluant. Fractions were collected from each run so that the maximum concentration at the apex of the elution profile could be measured. While the concentration of the substrates in the reactor was dynamic, measurement of the concentration at the peak of the elution profile provided a convenient means of comparing each of the runs. This also turned out to be an excellent means of predicting potential throughput under equilibrium concentration conditions (vide infra).

Photoreactor A (white LED) resulted in very low conversion for the two runs, and the results consisted primarily of recovered *n*-humulone **1a** (entries 1 and 2). The first run (entry 1) consisted of a 5 mL injection of a 50 mM solution of **1a** in ethanol. Due to the diffusion of the sample in the photoreactor, the product was collected over a total eluted volume of 50 mL. The maximum eluant concentration of 11.5 mM was only one-fifth of the injected sample concentration. For the second attempt (entry 2), a 50 mL injection of a 4.2 mM sample was introduced to the reactor followed by a 120 mL flush of ethanol to collect the entire sample. Using the larger injection, we were able to achieve a steady-state concentration of 4.2 mM in the reactor. A small increase in conversion to 6% was observed with a flow rate of 0.1 mL/min and a residence time of nearly 9 h. Although the white LEDs did have a broader range of spectral emission, they did not have significant emission at the lower wavelengths of the visible spectrum.

**Figure 4:**
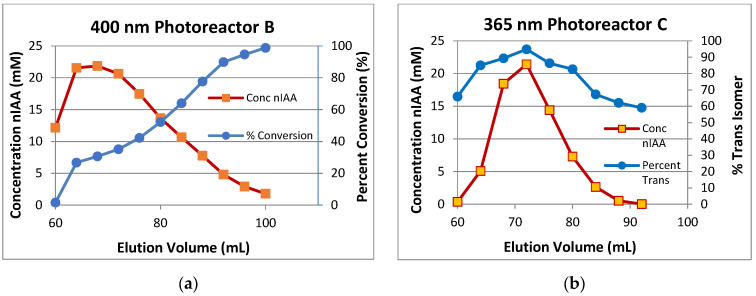
Reaction concentration of eluted fractions from photoreactors B and C: (**a**) elution profile of reaction concentration and percent conversion of a 5.0 mL injection of 0.1 M **1a** to photoreactor B; reaction concentration based on combined **1a** and product **2a**; (**b**) elution profile of total concentration of nIAA (**2a** and **3a**) and the percent trans isomer **2a** from a 5.0 mL injection of 0.1 M **1a**.

Photoreactor B used UVA LEDs and exhibited narrow spectral emission, with a peak at 400 nm. A 1.0 mL injection of 0.1 M **1a** resulted in a maximum eluant concentration of 3.5 mM, a 95% conversion to **2a** and an isolated yield of 83%. Increasing the injection amount to 5 mL (entry 4) resulted in an increase in the maximum eluant concentration and a lower conversion of only 54%. Analysis of individual 4 mL fractions demonstrated the impact of reaction concentration and potential conversion to product (Figure 4a). Maximum concentration was observed after elution of 70 mL of solvent and showed only 35% conversion. As the concentration of substrate and product continued to decrease, the conversion improved. Combined collection of all fractions (entry 4) showed 54% conversion. By decreasing the flow rate to 0.25 mL/min (entry 5) and maintaining the max. eluant concentration at 21 mM, we were able to achieve a nearly complete conversion of 95%, with an 83% yield of isolated product **2a**. In an effort to obtain higher throughput of material, we constructed photoreactor C using UVA 365 nm LEDs. This device had a higher overall wattage compared to reactors A and B and required water-cooled aluminum plates to obtain the desired reaction temperature of 25 °C. Excellent conversion was obtained for the higher maximum eluant concentrations of 24.9 and 21.4 mM for entries 6 and 7, respectively. However, use of reactor C led to significant epimerization of **2a** to give the cis diastereomer **3a** as a side product. Analysis of the fractions for the 200 min residence time run (entry 7) showed the most epimerization as occurring at the beginning and end of the run (Figure 4b). Presumably, higher reaction concentration provided more absorption of the photons by the substrate and protection of the product from epimerization. Maximum concentration of the eluant at 72 mL gave only 4% epimerization, while at 90 mL, near the end of the run, we observed 40% epimerization to **3a**. The combined fractions for the 0.25 mL/min run with photoreactor C gave a final mixture of **2a** and **3a** in a four-to-one ratio. The epimerization observed with photoreactor C was somewhat surprising in view of the report of Sharpe and Omrod [44] for the initial report of photochemical preparation of **2a** using a filtered medium-pressure Hg-lamp with maximum emission of 360 nm. The sensitivity of isohumulone **2a** to further photochemical reactions undoubtedly resulted in inconsistent and lower yields from batch reactors.

Based on the results from photoreactors A–C, it appeared that a higher wattage UVA 400 nm reactor would provide complete reaction with shorter residence times. Photoreactor D (395 nm, 42 W) was constructed to test this premise. We were pleased to find that the 1.0 mL/min run with a maximum eluant concentration of 22 mM (entry 8) provided complete conversion to product **2a** with an isolated yield of 97%. By comparison, photoreactor C provided only 54% conversion under identical conditions (entry 4). Longer residence times for reactor D did not result in significant amount of side products (entry 9, 95% conversion) showing the improved results from the use of narrow-wavelength 395 nm LEDs.

**Figure 5:**
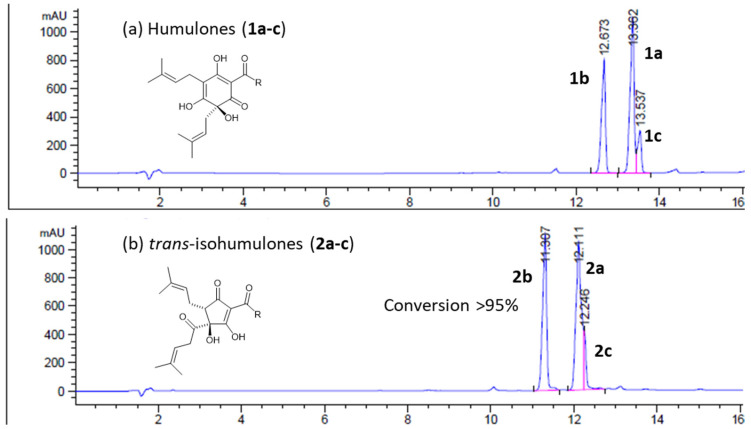
Chromatographic conditions listed in Section 3.1. HPLC analysis of homologs of (**a**) humulones **1a**–**c** and (**b**) trans-isohumulones **2a**–**c**. R group is isopropyl (**2a**), isobutyl (**2b**) and sec-butyl (**2c**). Final mixture ratio of trans-isohumulones **2b**:**2a**:**2c** is 40:51:9.

Determination of the throughput for synthesis of isohumulones **2a**–**c** was determined by introduction of a sufficient volume of the humulone **1a**–**c** solutions to obtain an equilibrium concentration of substrate in photoreactors B and D (Table 2). As we had seen from our 1–5 mL test reactions, significant diffusion was observed during the course of the reaction for these small volume injections. By increasing the sample introduction to 50 mL volume, we were able to obtain a constant concentration of substrate in the reactor. Experiments that resulted in quantitative conversion of humulones to isohumulones are shown in Table 2. Complete reactions were obtained with photoreactor B, using a 1.0 mL/min flow rate with a 6.6 mM reaction concentration (entry 1). The higher reaction concentration of 23 mM required a lower flow rate of 0.25 mL/min for complete reaction (entry 2). Comparable throughput for reactor B was seen for these two runs, of 0.143 g/h and 0.125 g/h, respectively. As expected from our test runs, a higher throughput was obtained with the higher-wattage photoreactor D, providing 0.434 g/h of isohumulone **2a** with residence time of just 50 min (entry 3). The homolog co-trans-isohumulone **2b** was prepared from co-humulone **1b** using photoreactor B with a non-optimized throughput of 0.104 g/h (entry 4). Mixtures of trans-isohumulones **2a**, **2b** and **2c** (isobutyl, isopropyl and sec-butyl, respectively) are commonly used as analytical HPLC standards for quantization of these bitter components in beer and hop teas [54]. Reported methods obtain these compounds by fractional crystallization of trans-isohumulone salts from isomerized hop extracts. Using a mixture of humulones **1a**–**c** obtained from hop extract, we obtained trans-isohumulones **2a**–**c** by flow photochemistry with a throughput of 0.391 g/h (Figure 5). As we observed for the isomerization of the individual humulones **1a** and **1b**, excellent conversion and purity were obtained for the trans-isohumulone mixtures of **2a**–**c**. This isohumulone mixture could be used directly as an analytical standard or converted to the dicyclohexylamine salt for long term storage.

## 3. Materials and Methods

### 3.1. General Methods

Commercial reagents of high purity were purchased (Millipore Sigma, St. Louis, MO, USA and Fisher Scientific, Hampton, NH, USA) and used without further purification. The *n*-humulone (**1a**), *co*-humulone (**1b**), mixture of humulones (**1a**–**c**), trans-isohumulones (**2a**–**c**) and cis-isohumulones (**3a**–**c**) were prepared as previously described [9]. Deionized water used for chromatography was purified by means of a Milli-Q Gradient A10 system (Millipore, Burlington, MA, USA). IR spectra were obtained by FTIR-ATR and reported in reciprocal wavenumbers (cm^−1^). ^1^H NMR spectra were referenced to residual CHCl_3_ (7.27 ppm). ^13^C NMR spectra were referenced to the center line of CDCl_3_ (77.2 ppm). Reverse-phase HPLC analysis was conducted with an Agilent 1200 system equipped with UV detection. Mobile phase solvents were prepared from HPLC grade 0.1% TFA in H_2_O (Mobile Phase A) and 0.1% TFA in CH_3_CN (Mobile Phase B). A Poroshell 120 EC-C18 column (3.0 mm id × 150 mm; 2.7 um particle size) was employed, using a flow rate of 0.4 mL/min and a gradient of 50%A/50% B from 0–2 min, and 50% B to 100% B from 2–12 min, followed by a hold of 100% B for 8 min. Preparative reverse-phase chromatography was carried out using a C18 column (10 um particle diameter; 250 mm × 20 mm i.d.) and isocratic mobile phases consisting of mixtures of HPLC-grade methanol and H_2_O at a flow rate of 10 mL/min. Details as to the construction of the flow photoreactors A–D are provided in the Supplementary Materials.

### 3.2. Continuous-Flow Photoreactor System

The continuous-flow photoreactor system consisted of a J-KEM programmable dual position syringe pump (KEMV6-2200-PC, J-KEM Scientific, St. Louis, MO, USA, www.JKEM.com), custom-built flow photoreactors and a Gilson fraction collector FC-204 (Gilson, Middleton, WI, USA, www.gilson.com). The J-KEM dual pump was equipped with 5 mL syringes connected to the flow reactor with Teflon tubing (1/16” od × 0.030” id). Each of the syringe pumps was equipped with a four-place switching valve to allow continuous-flow operation. A step-up Swagelock union was used to connect the Teflon tubing to the THV tubing (1/4” od × 5/32” id) of the photoreactor. The output of the photoreactor was connected directly to the robotic arm of the Gilson fraction collector. The reaction temperature of the photoreactors was 25–30 °C for all runs and was controlled by either a fan (photoreactors A and B) or a water-cooled aluminum plate (reactors C and D).

**Figure 6:**
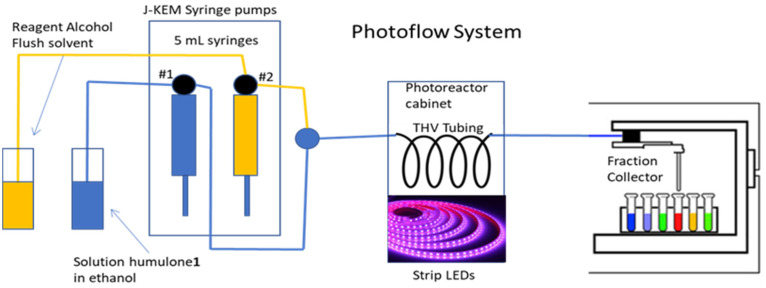
Schematic of continuous-flow photoreactor system.

Each reaction was carried out by using the dual syringe pumps to sequentially add the starting material from pump 1; this was followed by a complete flush of the system with the carrier solvent using pump 2 (Figure 6). Each pump was equipped with a four-way solenoid valve for flexibility in choosing either pump for the introduction and delivery of the solutions. Denatured alcohol or ethanol was used for preparation of substrate solutions and as the carrier solvent. The photoflow reactions can be described as analytical (Table 1) or preparative runs (Table 2). The flow system photoreactor was allowed to equilibrate to the proper flow rate and temperature prior to each run, such that the tubing in the photoreactor was devoid of any gas bubbles. For the analytical runs, a 1–10 mL solution of the humulone starting material in ethanol was introduced, followed by a complete flush of the system with ethanol or denatured alcohol. Fractions of 4 mL each were collected from each experimental run. Due to the diffusion of the reaction mixture during the flow reaction, a significant dilution effect was observed for the collected fractions. Introduction of a 5.0 mL solution of 0.10 M *n*-humulone **1a** in ethanol resulted in fractions with the maximum concentration of product **2a**, 0.021–0.027 M (Table 1. Entries 4–9). Introduction of 5 mL solution of starting material resulted in collection of product mixtures with a total volume of 50–60 mL. Concentrations of product and residual starting material were determined for each fraction by HPLC comparison of the product peak with a standard curve of analytically pure **2a**. The product and standard curve were determined based on absorption at 231 nm. Fractions obtained from each run were subsequently combined, concentrated in vacuo and reconstituted to a known volume for determination of product yield by HPLC and evaluation of purity by 1H NMR.

Preparative runs were carried out by the introduction of 50 mL of a solution of humulone **1** in ethanol at the appropriate concentration and flow rate (Table 2) followed by subsequent flush of the system with 125 mL of ethanol. The collected solution was concentrated in vacuo to provide the final product.

### 3.3. Synthesis of Trans-n-Isohumulone (***2a***)

A solution of 534 mg (1.13 mmol) *n*-humulone phenylenediamine salt in 30 mL of methylene chloride was prepared. The organic solution was washed twice with 3N HCl and washed once with water. The organic extract was dried with Na_2_SO_4_ and concd to give the free acid **1a** as a white solid. A solution of **1a** in ethanol was prepared and the final volume adjusted to provide a stock solution concentration of 0.020 M. Concentration of **1a** was confirmed by HPLC comparison with the analytically obtained standard curve (231 nm). Photoreactor D was equilibrated to 25 °C at a flow rate of 1.0 mL per minute with the ethanol carrier/flush solvent. Using syringe pump 1, 50 mL of the stock solution was introduced to the flow reactor. The dual pump system was programmed to follow the introduction of the stock solution with a flush of 125 mL of reagent alcohol from syringe pump 2. The collected fractions were concentrated in vacuo to give 340 mg (93%) of an off-white solid (95% pure by HPLC and 1H NMR). Recrystallization from methanol–water gave 268 mg (74%) of a crystalline, white solid as the free acid of trans-*n*-isohumulone **1a**; mp 64–65 °C (lit mp 63 °C [55]), HPLC retention time 12.13 min (98% UV 235); ${\left[a\right]}_{D}^{19}$ = −9.2 (EtOH, c = 2.5); lit [43] ${\left[a\right]}_{D}^{28}$ = −7.4° (MeOH); ^1^H NMR (CDCl_3_, 300 MHz): δ 11.50 (brs, 2H), 5.19 (t, 1H, *J* = 6.4 Hz), 5.13 (t, 1H, *J* = 6.3 Hz), 3.31 (m, 2H), 3.04 (dd, 1H, *J* = 6.0 Hz, 9.6 Hz), 2.71 (d, 2H, *J* = 7.0 Hz), 2.56 (m, 1H), 2.33 (m, 1H), 2.14 (m, 1H, *J* = 6.8 Hz), 1.73 (s, 3H), 1.68 (s, 3H), 1.56 (s, 3H), 1.53 (s, 3H), 0.96 (t, 6H, *J* = 7.6 Hz); ^13^C NMR (CDCl_3_): δ 206.9, 205.0, 197.8, 195.7, 136.2, 134.7, 120.1, 114.4, 110.3, 90.7, 55.3, 44.4, 38.7, 26.5, 26.4, 25.7, 25.6, 22.6, 22.3, 18.1, 17.9; HRMS (ESI−): *m*/*z* calcd for C_21_H_29_O_5_ (M − 1)^−1^: 361.2022; found: 361.2023 (+0.2 ppm).

### 3.4. Synthesis of Trans-co-Isohumulone (***2b***)

Photoreactor B was equilibrated to a temperature of 25 °C and a flow rate of 0.25 mL/min with reagent alcohol. Using syringe pump 1, a solution of 330 mg (0.95 mmol) of *co*-humulone **1b** in 50 mL reagent alcohol was introduced into the flow reactor followed by 125 mL of reagent alcohol from syringe pump 2. The eluant from the photoreactor was collected and concentrated in vacuo to afford 250 mg (76%) of **2b** as a light-yellow oil.

HPLC retention time 11.40 min (98% UV 235); ${\left[a\right]}_{D}^{24}$ = −4.4 (EtOH, c = 1.0); ^1^H NMR (CDCl_3_, 300 MHz): δ 5.19 (t, 1H, *J* = 6.4 Hz), 5.12 (t, 1H, *J* = 5.9 Hz), 4.10 (brs, 1H), 3.48 (m, 1H), 3.29 (d, 2H, *J* = 5.9 Hz), 3.04 (dd, 1H, *J* = 9.2, 6.3 Hz), 2.57 (m, 1H), 2.29 (m, 1H), 1.72 (s, 3H), 1.68 (s, 3H), 1.56 (s, 3H), 1.52 (s, 3H), 1.17 (d, 3H, *J* = 7.0 Hz), 1.13 (d, 3H, *J* = 7.0 Hz); ^13^C NMR (CDCl_3_): δ 206.9, 205.0, 203.4, 195.3, 136.2, 134.9, 120.0, 114.4, 109.1, 90.4, 54.7, 38.8, 34.6, 25.7, 25.6, 23.4, 18.1 (2C), 17.9 (2C); HRMS (ESI−): *m*/*z* calcd for C_20_H_28_O_5_ (M − 1)^−1^: 347.1864; found: 347.1873 (−2.5 ppm).

### 3.5. Synthesis of Mixture of trans-n,co,ad-Isohumulones (***2a***–***c***)

A mixture of homologs of humulone-phenylenediamine salts (52% *n*-humulone, 34% *co*-humulone, 14% *ad*-humulone) was prepared from CO_2_ hop extract [9]. A solution of 463 mg of a humulone-phenylenediamine salt in 30 mL of methylene chloride was washed twice with 3N HCl, followed by a single wash with water. The organic layer was dried with Na_2_SO_4_ and concd in vacuo to afford 332 mg (0.94 mmol) of the free acids as a viscous semi-solid. HPLC retention times in minutes were 12.67 (36%, **1b**), 13.38 (51%, **1a**) and 13.54 (13%, **1c**) (Figure 5a).

Photoreactor D was equilibrated to a temperature of 25 °C and a flow rate of 1.0 mL/min with ethanol. Using syringe pump 1, a 0.17M solution of **1a**–**c** in 50 mL of ethanol was introduced to the photoreactor, followed by 110 mL of ethanol from pump 2. The eluant from the photoreactor was collected and concentrated in vacuo to afford 327 mg (98%) of **2a**–**c** as a light-yellow oil. HPLC retention times in minutes were 11.31 (40%, **2b**), 12.11 (51%, **2a**) and 12.45 (9%, **2c**) (Figure 5b). A stable amine salt was prepared by dissolving the product in 15 mL of ethanol; this was followed by the addition of 0.30 mL of dicyclohexylamine. The cooled solution was treated with water and placed in a freezer overnight. The resultant crystalline salt was collected, washed with ethanol–water and dried, to afford 410 mg (82%) of a white, crystalline solid; mp 156–160 °C); ${\left[a\right]}_{D}^{24}$ = +28.6 (EtOH, c = 1.0).

### 3.6. Synthesis of Dehydrohumulinic Acid (***5***)

A solution of 180 mg of *n*-humulone **1a** dissolved in 3 mL of methanol was introduced into a microscale photochemical reactor equipped with a water-cooled medium-pressure Hg-lamp (Sigma-Aldrich (St. Louis, MO, USA), www.sigmaaldrich.com, cat # Z214558). The solution was irradiated for 24 h and subsequently concentrated in vacuo to give viscous oil. The product was purified by reverse-phase C18 chromatography with 50% methanol–water as the eluant. The eluant was concentrated and the aqueous mixture extracted with methylene chloride. Combined extracts were dried with Na_2_SO_4_ and concd in vacuo to give dehydrohumulinic acid **5**. The spectroscopic data are in full agreement with those reported in the literature [46]. HPLC retention time 9.31 min (98% UV 280); HRMS (ESI−): *m*/*z* calcd for C_15_H_20_O_4_ (M − 1)^−1^: 263.1289; found: 263.1289 (−0.2 ppm).

## 4. Conclusions

In summary, we have developed the first flow photochemical method for scalable, high yield isomerization of humulones to isohumulones. The continuous-flow approach avoids the inconsistent yields and conversions previously observed from batch photochemical reactions. Analysis of the flow reactions using different wavelength and power reactors allowed identification of side products, including epimerization of trans-isohumulones to cis isomers and potential hemolytic cleavage of the C4 side chain to give dehydrohumulinic acid. Elimination of these and other decomposition products was achieved by selection of optimal emission of the light source and control of reaction residence time. The custom-built photoreactors can be easily constructed using low-cost, commercially available strip LEDs and simple holders to provide cooling and protect users from the UV light. We were also able to demonstrate the utility of using small injections in the flow system to evaluate the potential for throughput under equilibrium constant conditions. This can be applied to any flow chemistry system and is particularly useful when evaluating optimal reactor conditions with limited amount of substrate material.

## Figures and Tables

**Table 1:** Flow photochemical isomerization of *n*-humulone (**1a**) to trans-*n*-isohumulone (**2a**).

Photoreactor	Entry	Flow Rate (mL/min)	Conc 1a (mM)	Injection Volume (mL)	Residence Time (min)	Max. Eluant Conc (mM) ^a^	Conversion (%) (% Yield 2a) ^b^
A (white LED, 18 W)	1	1.0	50	5	50	11.5	2.3
	2	0.10	4.2	50	500	4.2	6.0
B (400 nm, 17 W)	3	1.0	100	1	50	3.5	95 (83%)
	4	1.0	100	5	50	21.9	54
	5	0.25	100	5	200	23.4	95 (83%)
C (365 nm, 46 W)	6	1.0	100	5	50	24.9	91 (**2a** and **3a**) ^c^
	7	0.25	100	5	200	21.4	95 (**2a** and **3a**) ^d^
D (395 nm, 42 W)	8	1.0	100	5	50	22	99 (97%)
	9	0.25	100	5	200	27	95
	10	2.0	100	10	25	33	50.7

^a^ Highest concentration of **1a** and **2a** observed in the collected fractions from the photoreactor. ^b^ Conversion determined by HPLC of the combined eluted product compared to a known standard curve. Isolated yields represent gravimetric determination of **2a**. ^c^ Product mixture of trans **2a** (94%) and cis**2b** (6%) isomers. ^d^ Product mixture of trans **2a** (79%) and cis **2b** (21%) isomers.

**Table 2:** Product throughput for isohumulones **2a**–**c** from photoreactors B and D.

Entry	Photoreactor	Product	Flow Rate (mL/min)	Conc (mM)	Throughput ^1^ (g/h)
1	B	2a	1.0	6.6	0.143
2	B	2a	0.25	23	0.125
3	D	2a	1.0	20	0.434
4	B	2b	0.25	20	0.104
5	D	2a-c	1.0	18	0.391

^1^ Throughput obtained from combined collection of eluant from introduction of a 50 mL solution of humulones **1a**–**c** to photoreactor B or D. Products obtained in >95% conversion and yield as determined by HPLC and 1H nmr.

## Data Availability

Data are contained within the article and Supplementary Materials.

## Supplementary Materials

The following supporting information can be downloaded at: https://www.mdpi.com/article/10.3390/molecules30051002/s1, Details for construction of the custom continuous-flow photoreactors, tables of individual flow synthesis experiments, synthetic details, preparation of humulone-phenylenediamine salts, and 1H and 13C NMR spectra can be found online.
