# Supplementary material for: Continuous-Flow Photochemical Isomerization of Humulones to Isohumulones
Source: Molecules. 2025 Feb 21;30(5):1002. doi: 10.3390/molecules30051002 (PMC11901516; doi:10.3390/molecules30051002)
Supplement: Supplementary file 1 [file molecules-30-01002-s001.zip › molecules-3475672-supplementary.pdf]

Continuous Flow Photochemical Isomerization of Humulones to Isohumulones

Bruce C. Hamper, Bradley Gallow, Gregory Giovine and Trevor Smith*

University of Missouri-St. Louis, Department of Chemistry & Biochemistry, 43 Benton Court, St. Louis, MO 63122, USA

hamperb@umsl.edu

Contents

1. Experimental Methods

Methods and Materials 3

2. Flow Photoreactors

Batch Photoreactor
Batch LED Photochemical Reactor 3
Figure S1. Construction of the LED photoreactor for batch reactions 4
Flow Photoreactors
Construction of Continuous Flow Photoreactors 4
Figure S2. Construction of aluminum spool as base for photoreactors 4
Table 1. Specifications of the photoreactors A-D. 5
Figure S3. Construction of LED strips and THV tubing to the aluminum spool 5
Construction of Safety Box for Photoflow Reactors 6
Figure S4. CAD diagram of the photochemical reactor box 7
Figure S5. Liquid cooling plates for photoreactor 8
Figure S6. Completed boxes for the photoreactors 9
Figure S7. Photoreactor boxes with the doors attached 10
Figure S8.Assembled photochemical reactor 11

3. Synthetic Methods

Batch Photochemical Synthesis of (-)-trans-*n*-isohumulone (**2a**) 12
Continuous PhotoFlow Synthesis of mixture of homologs:
co-,n-,ad-trans-iso-humulone dicyclohexylamine salt (2a-c) 13

4. References 13

5. Emission Spectra 14
Figures S9-S12: LED emission spectra

6. NMR Data 16
(-)-trans-*n*-isohumulone (**2a**) 16
(-)-trans-*co*-isohumulone (**2b**) 17

7. LCMS Data 18
General Methods 18
HPLC and Mass Spectra 19

1. Experimental Methods

Materials and Methods

Commercial reagents of high purity were purchased (Millipore Sigma, USA and Fisher Scientific, Hampton, NH) and used without further purification. Supercritical CO_2 hops extract (Batch AU-401) was obtained from Hopsteiner (S.S.Steiner, Yakima, Washington, USA). Deionized water used for chromatography was purified by means of a Milli-Q Gradient A10 system (Millipore, USA).IR spectra were obtained by FTIR-ATR and reported in reciprocal wavenumbers (cm^{-1}). ^1H NMR spectra were referenced to residual $CHCl_3$ (7.27 ppm) or DMSO (2.54 ppm). ^{13}C NMR spectra were referenced to the center line of $CDCl_3$ (77.2 ppm) or DMSO (40.4 ppm). Reverse phase HPLC analysis was conducted with an Agilent 1200 system equipped with UV detection. Mobile phase solvents were prepared from HPLC grade 0.1% TFA in H_2O (Mobile Phase A) and 0.1% TFA in CH_3CN (Mobile Phase B). A Poroshell 120 EC-C18 column (3.0 mm id x 150 mm; 2.7 um particle size) was employed using a flow rate of 0.4 mL/min and a gradient of 50%A/50% B from 0 – 2 min, 50% B to 100% B from 2 – 12 min followed by a hold of 100% B for 8 minutes. Preparative reverse phase chromatography was carried out using a C18 column (10 um particle diameter; 250 mm x 20 mm i.d.) and isocratic mobile phases of consisting of mixtures of HPLC grade methanol and H_2O at a flow rate of 10 mL/min.

The LED strip lights for reactors A and B were obtained from Superbright LEDs (www.superbrightleds.com) for the white light source (STN-A50K80-B6A-08B5M-24V) and UVA 400 nm light source (STN-BULV-B6A-08B5M-24V). LEDs for reactors C and D were obtained from Waveform (www.waveformlighting.com) for the UVA 365 (7021.65.5M) and UVA 395 (7021.95.5M), respectively. THV tubing used for the photoreactors was 0.25 in od x 5/32" id with a wall thickness of 0.047" (www.mcmaster.com; McMaster Carr #5737K41). Flow control for the photoreactors was obtained using a J-KEM programmable dual syringe pump (www.jkem.com; KEMV6-2200-PC) equipped with 5 mL syringes. Temperature of the apparatus was monitored using a J-KEM model 210 thermocouple.

2. Photochemistry Reactors

Batch LED Photoreactor

A photoreactor was constructed from a string of flexible light strip LEDs and a 40 mL capped vial. The light source consists of an LED light strip 70 cm in length by 8 mm wide with 42 individual UVA1 LEDs (superbrightleds.com; STN-BULV-B6A-08B5M-24V). Each LED (3528 chip, near UV) is rated at 80 mW for a total output of 3.36 W for the 42 LED string. Measurement of the emission spectrum shows a single band at 400 nm. A 24V DC power supply (CPS-24V-36W) was used with a CPS connector (CPS-F2ST) to the LED light strip. The completed assembly was equipped with a magnetic stirrer and a N_2 line.

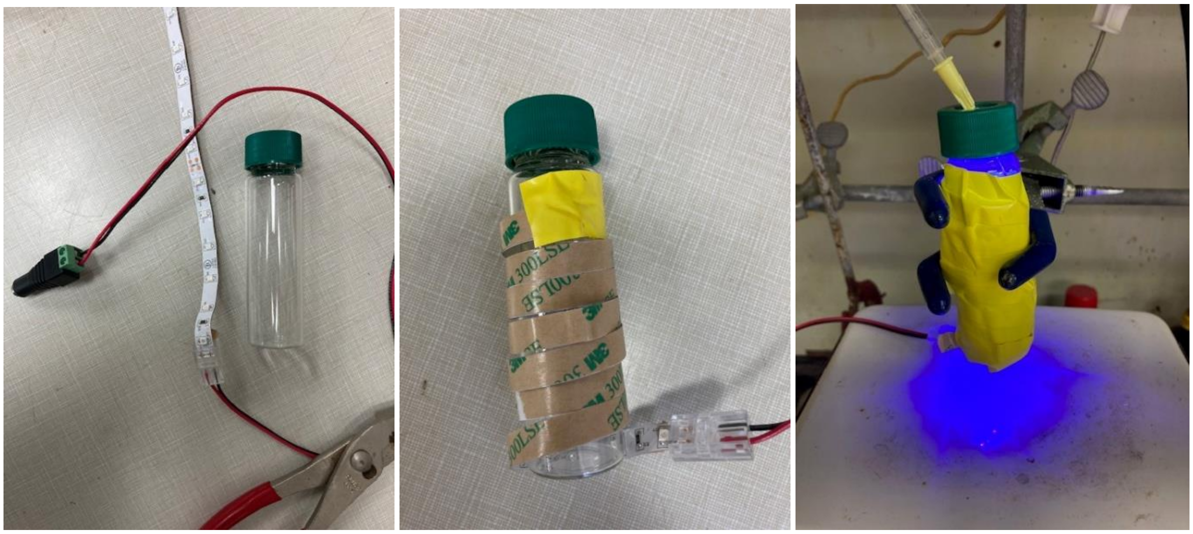

Figure S1. Construction of the LED photoreactor for batch reactions.

Construction of Continuous Flow Photoreactors

For our studies, we built four flow photoreactors (A, B, C, D) using an aluminum base, strip LEDs and UV transparent THV polymer tubing (Figure 2). THV tubing is very similar to FEP, but contains an additional vinylidene fluoride copolymer to provide greater tubing flexibility. The base was constructed from an aluminum cylinder (10 cm diameter x 10 cm h) that was milled to provide a lip at each end for alignment of the LEDs and THV tubing. LED strips with an adhesive backing were wound around the aluminum base followed by an aligned winding of the THV tubing. The width of the LED strips and the THV tubing were closely matched to allow the best possible alignment of the light source and tubing. As such, the entire length of tubing was in direct contact with the individual LED chips. The geometry of the device resulted in an average of 13 windings of THV tubing (440 cm length x 6.35 mm od x 4.0 mm id) and a void volume of 53-66 mL. The first two reactors A (white) and B (400 nm) had greater spacing between the LED chips resulting in lower overall wattage for the device (17-18 W). The aluminum cylinder provided a convenient design for cooling. A box was constructed with openings at the top and bottom of the box and a 12V computer fan for temperature control. These openings in the box aligned with the inner bore of the aluminum spool to allow efficient air flow. For reactors A and B, this provided sufficient cooling such that reactions were maintained below 30°C. Reactors C (Waveform-365 nm) and D (Waveform-395 nm) used LED strips with twice as many chips per length of the strip resulting in higher wattage devices (42 W). The higher wattage reactors led to greater heat production. In order to provide more efficient control of temperature, we devised a holder that used two liquid cooling plates consisting of copper tubing and aluminum. The copper tubing was attached to a recirculating laboratory chiller and provided precise control of the temperature for the photoreactors. The constructed box for the completed photoreactor provided both alignment of the cooling plates

for temperature control and a suitable container for protection of the operator from the UVA light source.

Using stock aluminum tubing (12 cm od x 9.5 cm id) a set of Al spools were prepared in our machine shop. The stock tubing was cut to 12 cm lengths and a center section was milled down 0.9 cm to give the shape of a spool. A 0.9 cm ridge was kept at each end of the spool to provide stability and alignment of the LEDs and tubing. A metal bar was added (12 cm x 1 cm x 0.3 cm) to allow a means of securing the tubing of final constructed photoreactor.

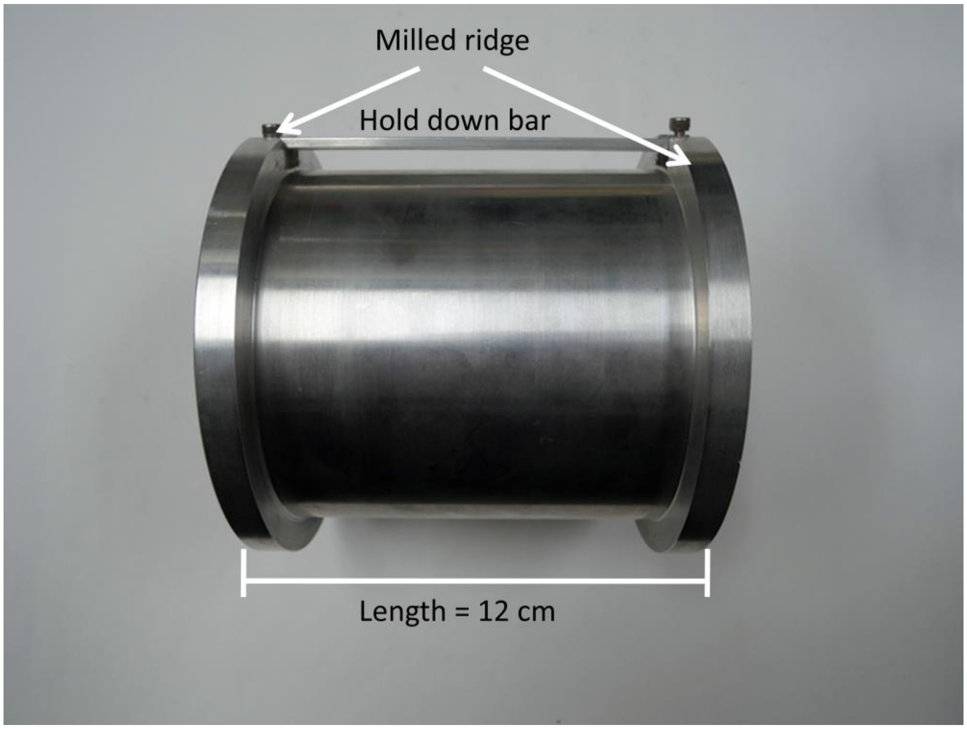

Figure S2. Construction of aluminum spool as base for photoreactors.

Each reactor spool was wound with LED strip lights that had an adhesive backing. Depending on the width for the LED strip lights, between 9 and 11windings were applied to the aluminum spool. Four types of LEDs were used. For the Superbright LEDs (superbrightleds.com; STN-BULV-B6A-08B5M-24V) we obtained 11 turns on the spool. The waveform LEDs (waveform.com; Part # 7021.95.5M) were wider and resulted in 9 windings on the Al spool (Table 1). Superbright LEDs: 9 mm width; 6 LEDs/10 cm; 24V DC. Waveform realUV™ LED Strip Lights: 10 mm width; 12 LEDs/10 cm; 12V DC

Table 1. Specifications of the photoreactors A-D.

PhotoReactor	A	B	C	D
LEDs	Superbright White	Superbright 400	Waveform 365	Waveform 395
Measured wavelength	Visible-broad spectrum	400 nm	368 nm	396 nm

Length of LED strip	380 cm	360 cm	292.5 cm	290 cm
Number of LEDs	228	216	351	348
Total Wattage	18.24 W	17.3 W	42.1 W	41.8 W
Length of THV tubing	408 cm	442 cm	510 cm	442 cm
Volume	53 mL	57 mL	66 mL	57 mL

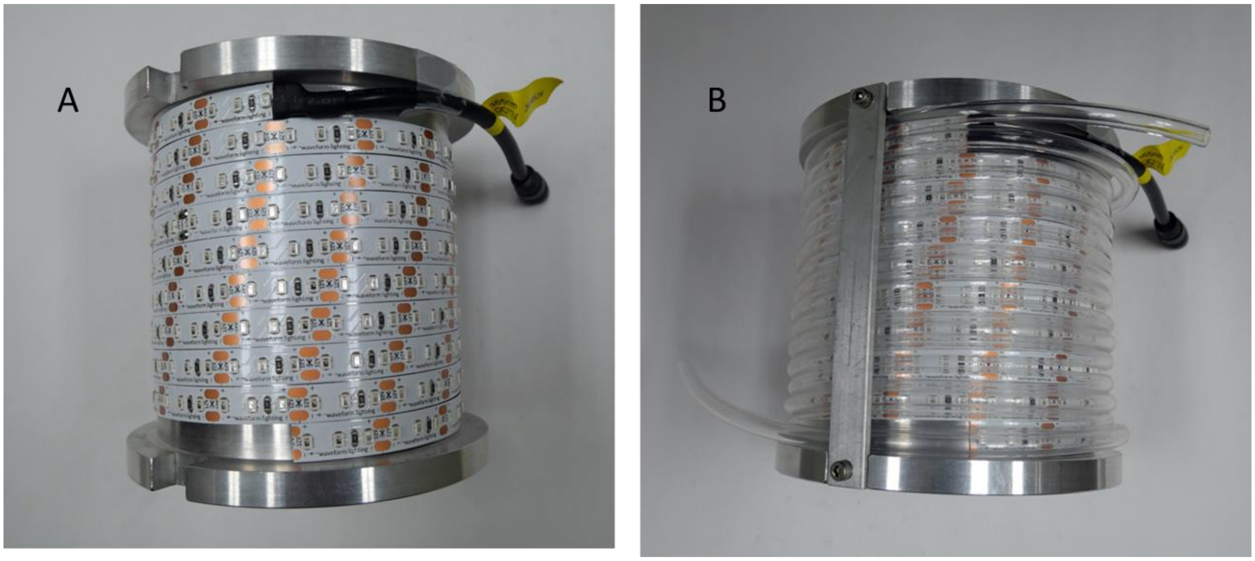

Figure S3. Construction of LED strips and THV tubing to the aluminum spool.

After applying the LEDs, the UV transparent THV tubing (0.25 in (6.3 mm) od x 5/32” (4.0 mm) id) was added. THV consists of a blend of three monomers; Tetrafluoroethylene, Hexafluoropropylene and Vinylidene Fluoride. By comparison, FEP is a copolymer of tetrafluoroethylene and hexafluoropropylene. The addition of vinylidene fluoride to THV improved the flexibility of the tubings; an important aspect for our applications using the aluminum spool. For similar dimension tubing, THV has a bend radius of 7.4 cm (0.29 in), while FEP has a bend radius of about 1.9 cm (0.75 in). UV transparency is similar for both THV and FEP. Depending how tightly the tubing was wound on the spool, between 12 and 15 turns were obtained. This resulted in total volumes for the reactors of 53 to 66 mL (table).

Construction of Safety Box for Photoflow Reactors

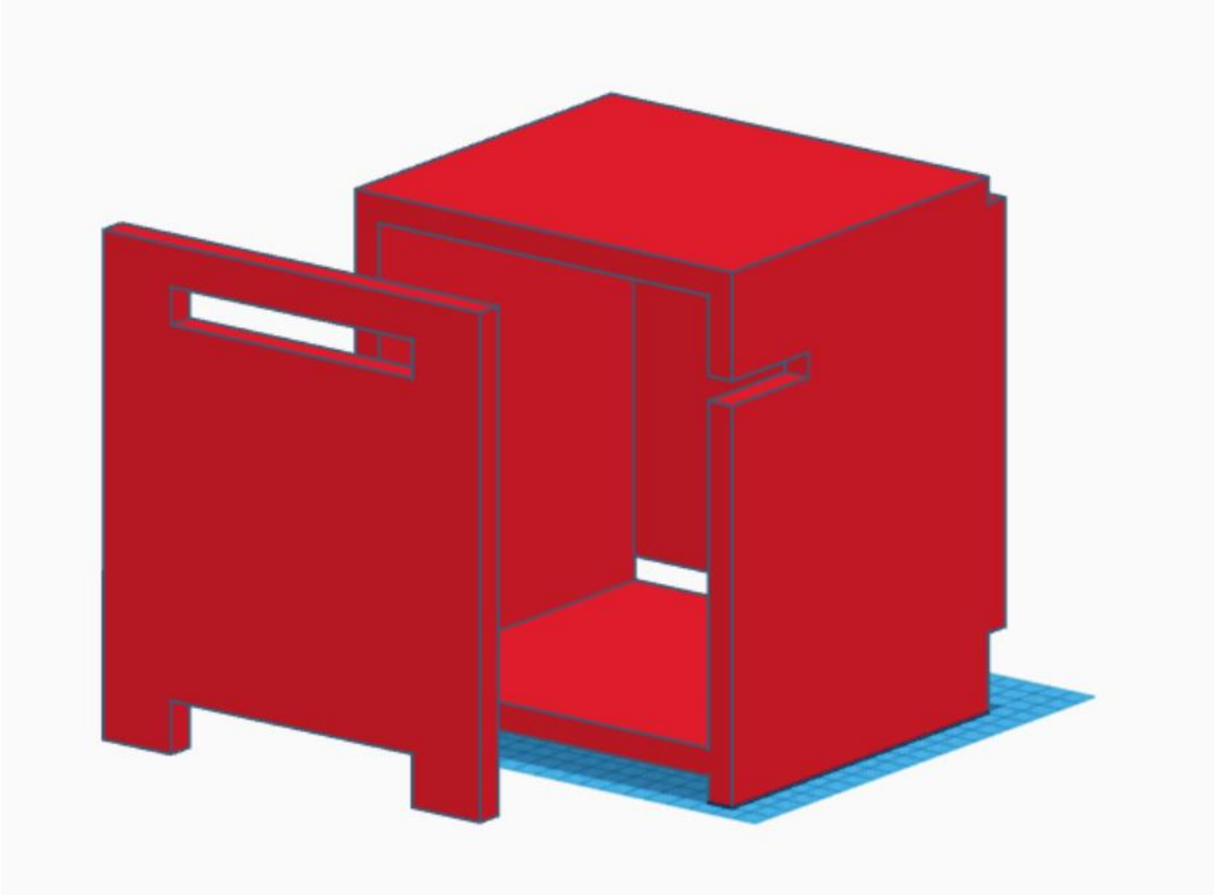

Figure S4. CAD diagram of the photochemical reactor box.

To provide cooling and suitable protection from UV light, a set of simple boxes were constructed. Using ½″ birch plywood two boxes were built; one for air cooling and one for use of water cooled plates. The box consisted of six parts:

Sides (2) – 6″ (15.2 cm) x 8″ (20.3 cm)

Top – 6″ (15.2 cm) x 6″ (15.2 cm)

Bottom - 6″ (15.2 cm) x 6″ (15.2 cm)

Back – 6″ (15.2 cm) x 7″ (17.8 cm)

Door - 6″ (15.2 cm) x 7″ (17.8 cm)

The box components were cut to size and, with the exception of the door, assembled using wood glue. Slots were added to accommodate the inlet and outlet of the tubing and LED power cord.

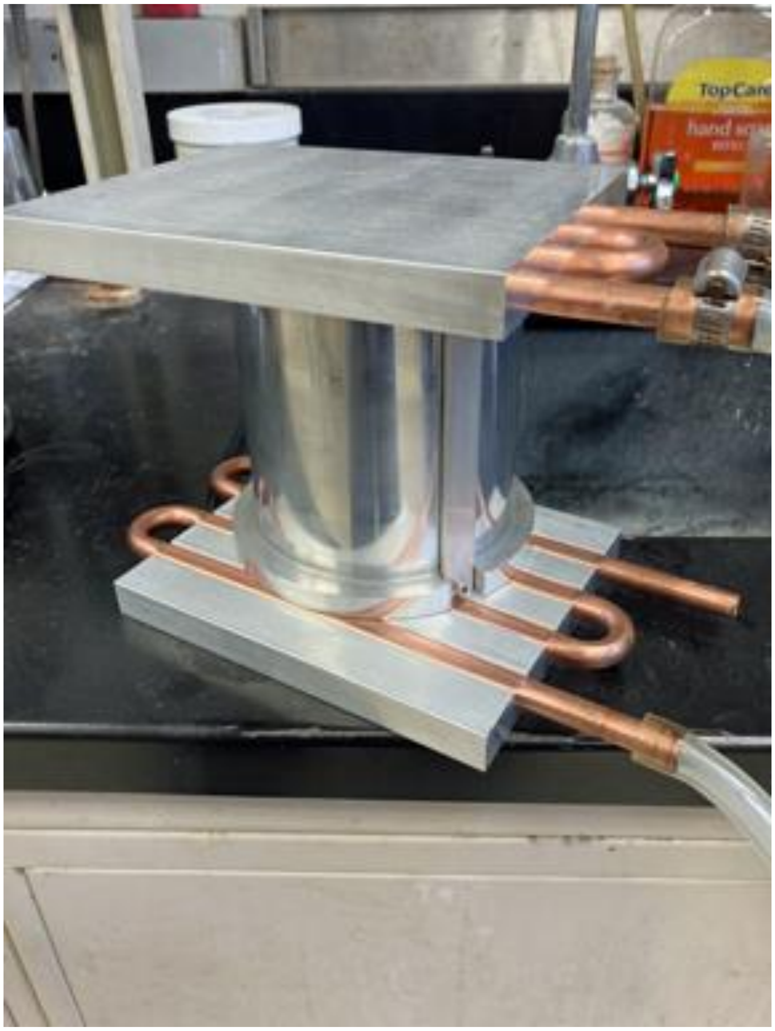

Figure S5. Liquid cooling plates for photoreactor

Two different means for cooling the reactors were investigated. Photoreactors A and B were of lower wattage and were adequately cooled with a standard 12V computer cabinet fan (Arctic Cooling P12 (120 mm case, 12 VDC, 0.16A; MicroCenter #579888)). The higher wattage photoreactors C and D required additional cooling capacity to maintain the reactions at room temperature. A set of two liquid cooling plates (Mouser Electronics, www.mouser.com; #984-ATS-TCP1001) attached to a chiller recirculation bath were used. With the chiller temperature set at 10 ^{o}C, the photoreactors C and D were maintained at temperatures of 22-25 ^{o}C.

Photoreactor D was constructed using Waveform lighting realUV™ LEDs (Part # 7021.95.5M). Manufacturer's data sheet for 395 nm strip gives power input of 4.8 W/ft (12V DC), a UV output of 1.1 W/ft and an efficiency of 22%.

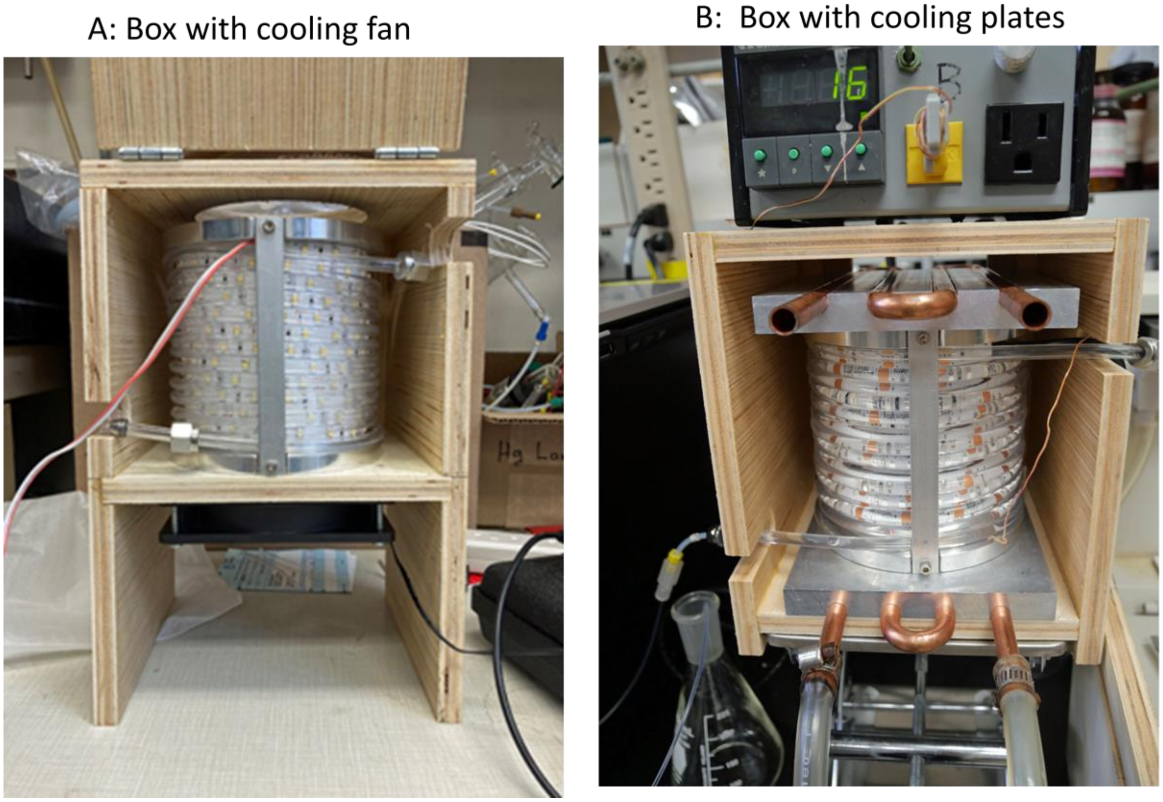

Figure S6. Completed boxes for the photoreactors. A) Cooling provided by a 12V computer cabinet fan. B) Cooling provided by a set of liquid cooling copper pipes imbedded in an aluminum block.

A: Box with cooling fan

B: Box with cooling plates

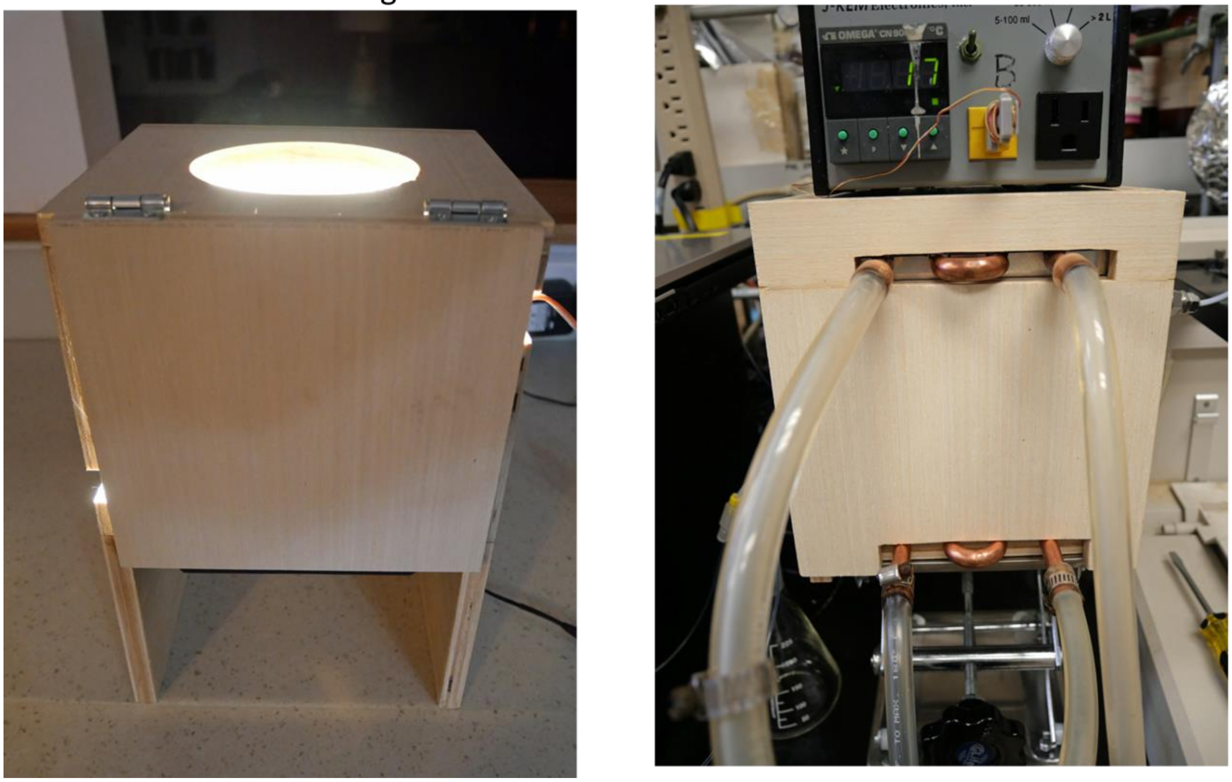

Figure S7. Photoreactor boxes with the doors attached

Tubing from pump: 1/16 x 0.03 ID Teflon tubing Fingertight fittings.

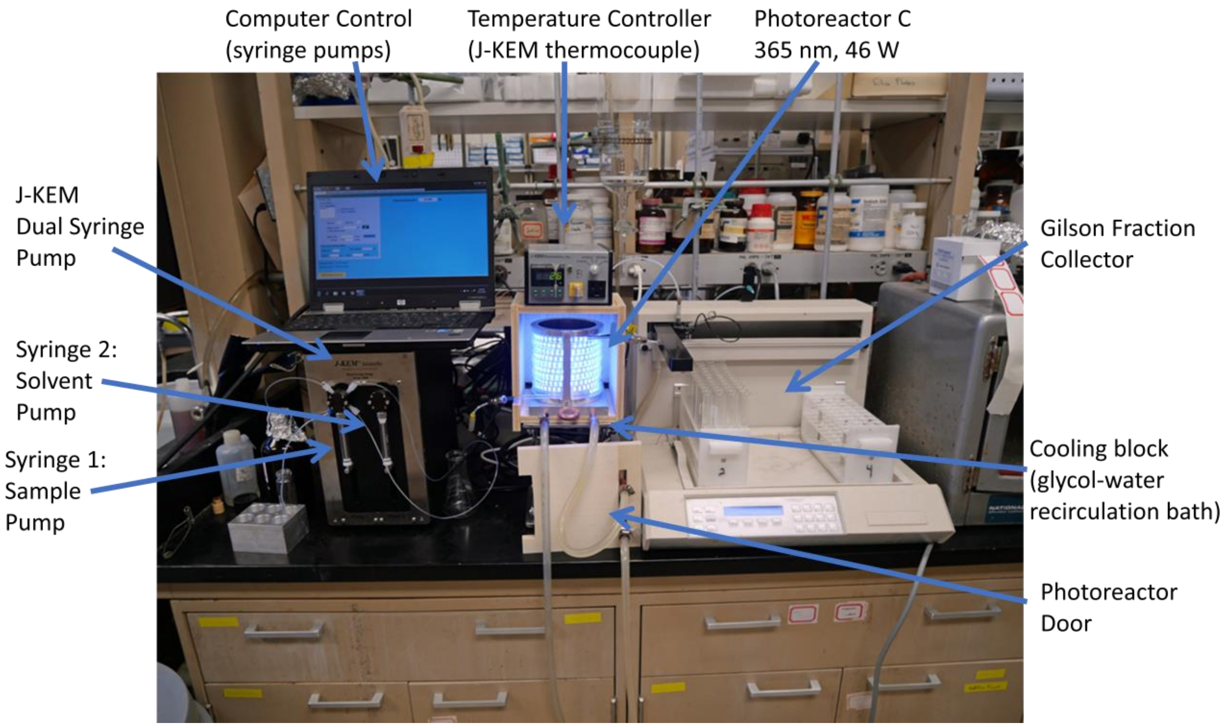

Figure S8.Assembled photochemical reactor station with computer control and fraction collector.

3. Synthetic Methods

Batch Photochemical Synthesis of *n*-trans-iso-humulone 2a.

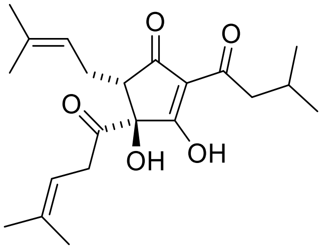

Attempts to carryout batch photosynthesis of **2a** on scale larger than 0.35 mmole led to low yields and low purity of final product. However, reasonable yield and recovery were obtained using the 40 mL batch photochemical vial equipped with 400 nm LEDs.

A solution of 155 mg (0.35 mmole) of *n*-humulone salt **1a-PDA** in 20 mL of CH_2Cl_2 was acidified with 10 mL of 1N HCl. The collected organic layer was concentrated to afford the free acid *n*-humulone **1a**. The resultant oil was dissolved in 10 mL of denatured alcohol and placed in the LED equipped photoreactor vial. After stirring for 8 hrs under exposure to the LEDs, the mixture was concd in vacuo to afford a light yellow oil. The oil was dissolved in hot isooctane and the solution placed in the freezer for 3 days. The product was collected by filtration, washed with cold isooctane and allowed to air dry to afford 90 mg (71%) of a yellow, crystalline solid.

mp 52-56 ^{o}C. (lit mp 63 ^{o}C [1])

HPLC (UV 280 nm): 12.32 min (98%).

UV (0.1%TFA in 80% CH_3CN/H_2O): $_{max}$ = 226, 281 nm;

$[a]_D^{23}$ = -5.0 (EtOH, c = 1.6); lit [2] $[a]_D^{28}$ = -7.4^{o} (MeOH).

FTIR (ATR): 3407 (m, br), 2925 (m), 1697 (s), 1626 (s), 1548 (s).

^{1}H NMR ($CDCl_3$, 300 MHz): 11.50 (brs, 2H), 5.19 (t, 1H, *J* = 6.4 Hz), 5.13 (t, 1H, *J* = 6.3 Hz), 3.31 (m, 2H), 3.04 (dd, 1H, *J* = 6.0 Hz, 9.6 Hz), 2.71 (d, 2H, *J* = 7.0 Hz), 2.56 (m, 1H), 2.33 (m, 1H), 2.14 (m, 1H, *J* = 6.8 Hz), 1.73 (s, 3H), 1.68 (s, 3H), 1.56 (s, 3H), 1.53 (s, 3H), 0.96 (t, 6H, *J* = 7.6 Hz).

^{13}C NMR ($CDCl_3$): 206.9, 205.0, 197.8, 195.7, 136.2, 134.7, 120.1, 114.4, 110.3, 90.7, 55.3, 44.4, 38.7, 26.5, 26.4, 25.7, 25.6, 22.6, 22.3, 18.1, 17.9.

MS (ESI-): *m/z* (%) 361 (M-1, 7), 198 (100).

HRMS (ESI-): *m/z* calcd for $C_{21}H_{29}O_5$ $(M-1)^{-1}$: 361.2022; found: 361.2023 (+0.2 ppm).

Continuous PhotoFlow Synthesis of mixture of homologs; *co-,n-,ad*-trans-iso-humulone dicyclohexylamine salt (2a-c).

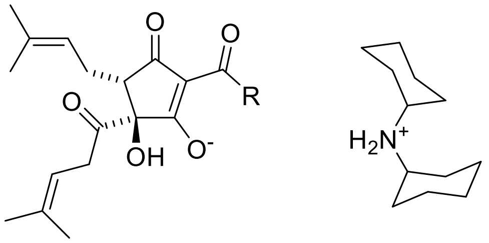

R = isobutyl, secbutyl, isopropyl

A mixture of homologs of humulone-phenylenediamine salts (52% n-humulone, 34% cohumulone, 14% adhumulone) was prepared from CO_2 hop extract [3]. A solution of 463 mg of the humulone-phenylenediamine salt in 30 mL of methylene chloride was washed twice with 3N HCl followed by a single wash with water. The organic layer was dried with Na_2SO_4 and concd in vacuo to afford 332 mg (0.94 mmol) of the free acids as a viscous semi-solid. HPLC retention times in minutes; 12.67 (36%, **1b**), 13.38 (51%, **1a**) and 13.54 (13%, **1c**)

A Photoreactor D was equilibrated to a temperature of 25 ^oC and a flow rate of 1.0 mL/min with reagent alcohol. Using syringe pump 1, a 0.17M solution of **1a-c** in 50 mL of reagent alcohol was introduced to the photoreactor followed by 110 mL of reagent alcohol from pump 2. The eluant from the photoreactor was collected and concentrated in vacuo to afford 327 mg (98%) of **2a-c** as a light yellow oil. HPLC retention times in minutes; 11.31 (40%, **2b**), 12.11 (51%, **2a**) and 12.45 (9%, **2c**). A stable amine salt was prepared by dissolving the product in 15 mL of ethanol, followed by addition of 0.30 mL of dicyclohexylamine. The cooled solution was treated with water and placed in a freezer overnight. The resultant crystalline salt was collected, washed with ethanol-water and dried to afford 410 mg (82%) of a white, crystalline solid.

mp 156-160 ^oC.

$[a]_D^{23}$ = + (EtOH, c = 1.1);

HPLC (UV 231 nm): 11.20 (**2b**, 39%), 12.02 (**2a**, 51%), 12.14 (**2c**, 9.5%).

Peak **2b**: HRMS (ESI-): *m/z* calcd for $C_{20}H_{27}O_5$ $(M-1)^{-1}$: 347.1866; found: 347.1871 (1.6 ppm).

Peak **2a/2c**: HRMS (ESI-): *m/z* calcd for $C_{21}H_{29}O_5$ $(M-1)^{-1}$: 361.2022; found: 361.2023 (+0.2 ppm).

4. References

1. Viriot, M.; Andre, J.; Niclause, M.; Bazard, D.; Flayeux, R.; Moll, M. Improvement of the bitterness of hops: Photoreactions of alpha acids. *J. Inst. Brew.* **1980**, *86*, 21-24.

2. Clarke, B.; Hildebrand, M. The isomerization of humulone; I. Isolation of photoisohumulone. *J. Inst. Brew.* **1965**, *71*, 26-36.

3. Hamper, B. C.; Campbell, H. J.; Luo, R.; Murphy, M.; Gleason, P.; Smith, T.; Jagan, R. Selective synthesis of deuterated cis- and trans-isohumulones and trans-isohumulinones. *Synthesis,* **2024**, *56*, 3206-3214.

5. **Emission Spectra from LED Photoreactors**

Spectral Measurement with Vernier Spec Optical Fiber (VSP-FIBER) and Vernier UV-VIS Spectrophotometer (VSP-UV) (www.vernier.com)

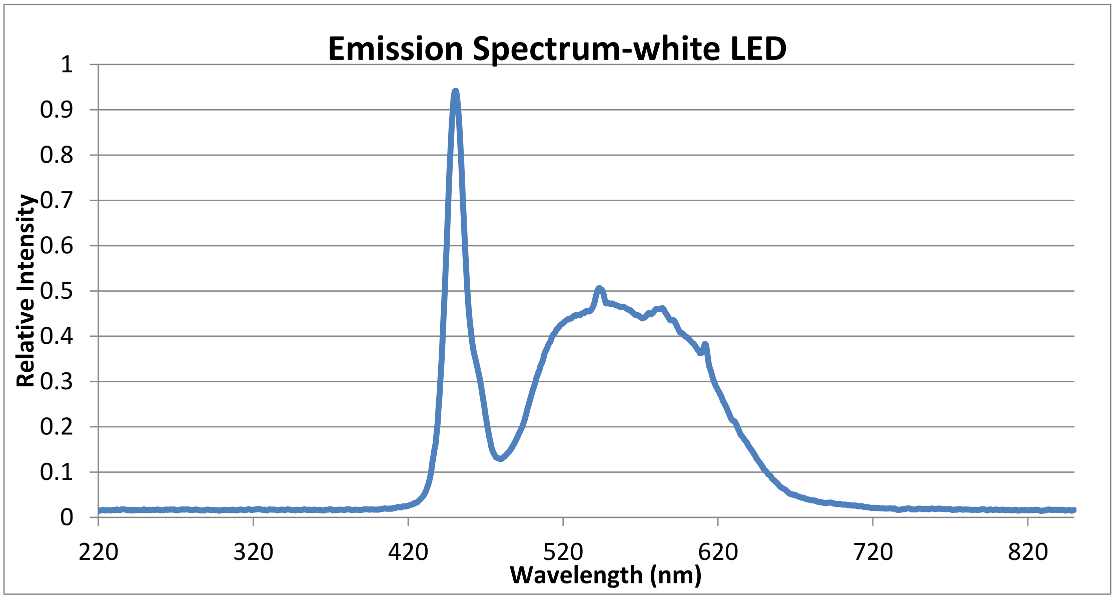

Figure S9. Emission spectrum from Photoreactor A (Superbright white LED). Maximum intensity observed at 450 nm with a broad band from 510 – 630 nm.

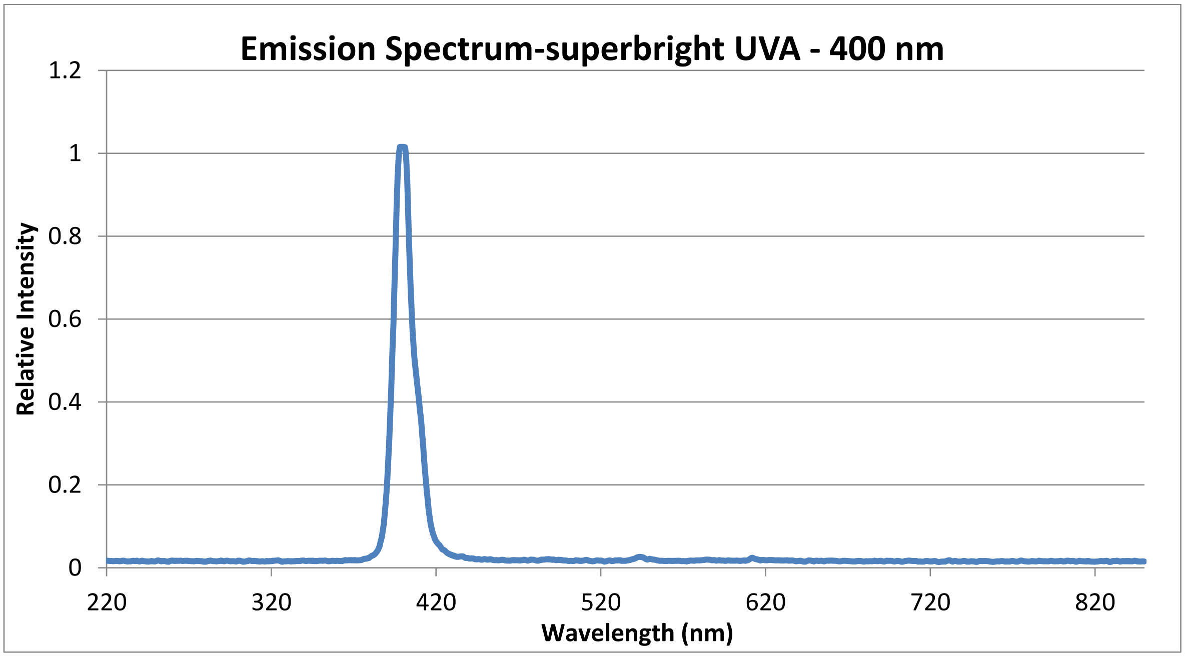

Figure S10. Emission spectrum from Photoreactor B (Superbright UVA1 LED; 3528 chip) with maximum emission at 400 nm.

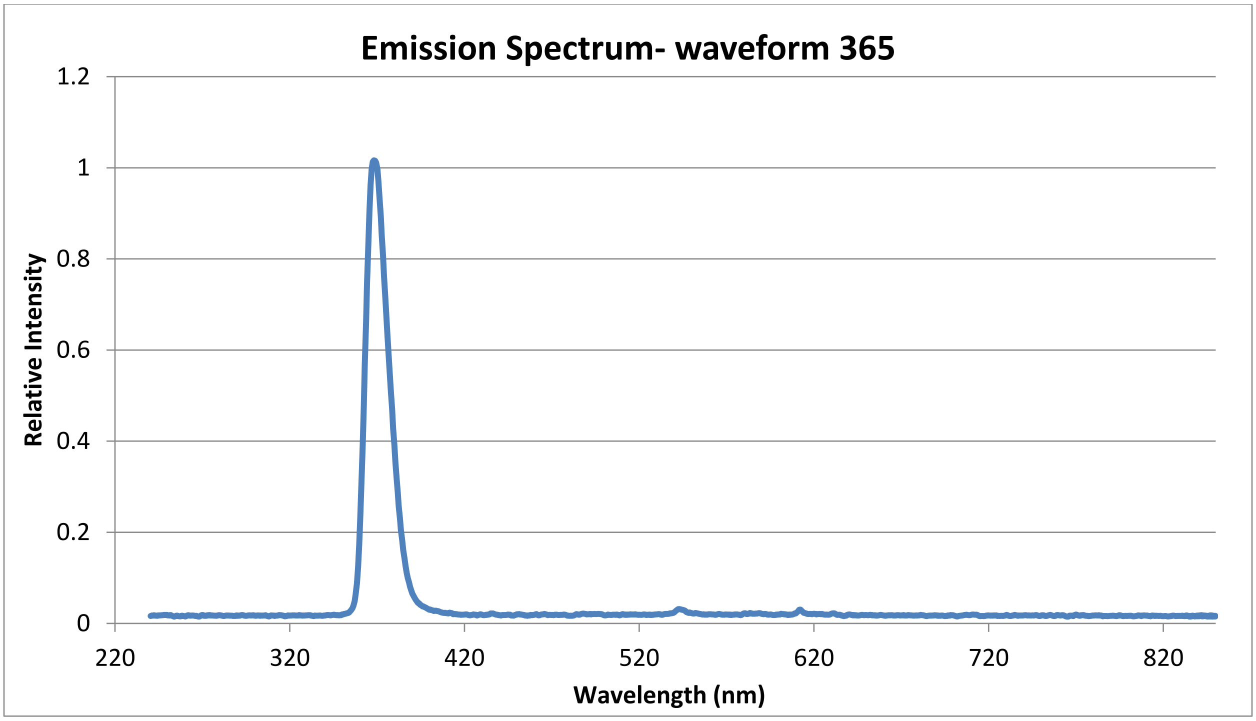

Figure S11. Emission spectrum from Photoreactor C (Waveform Lighting realUV™ LED) with maximum emission observed at 368 nm.

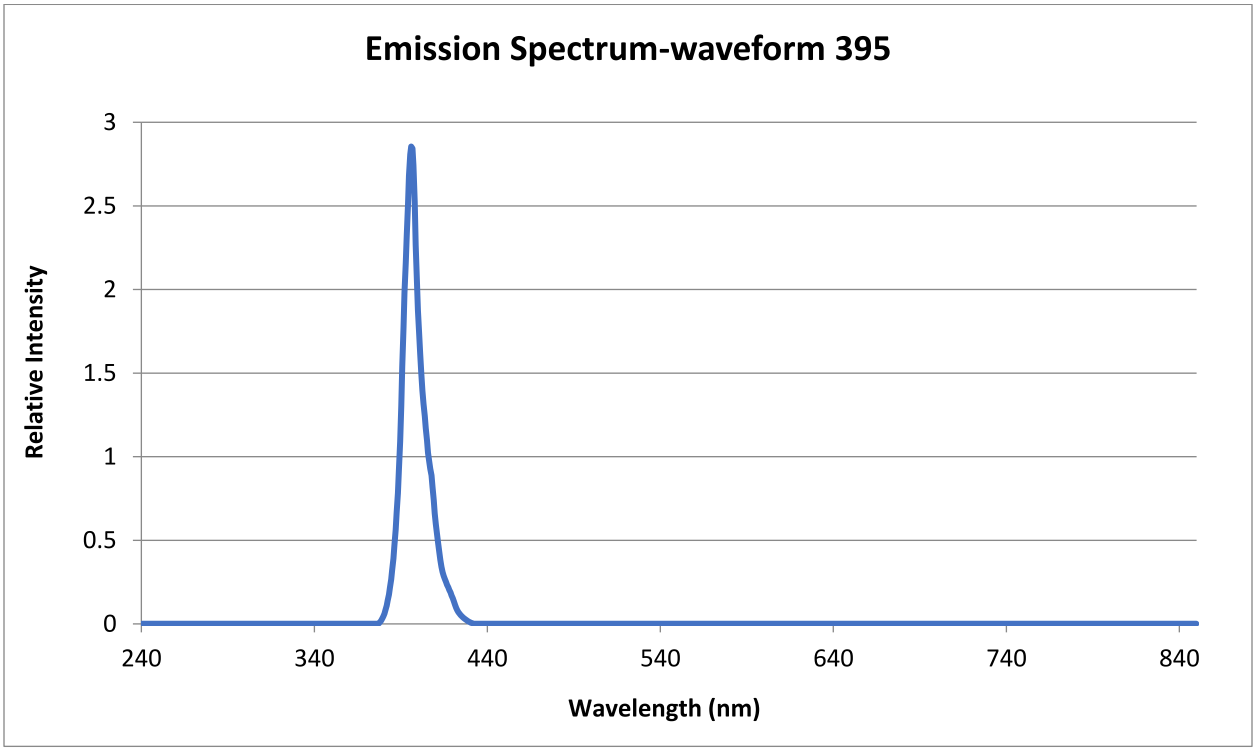

Figure S12. Emission spectrum from Photoreactor D (Waveform Lighting realUV1™ LED) with maximum emission observed at 396 nm.

6. NMR Data

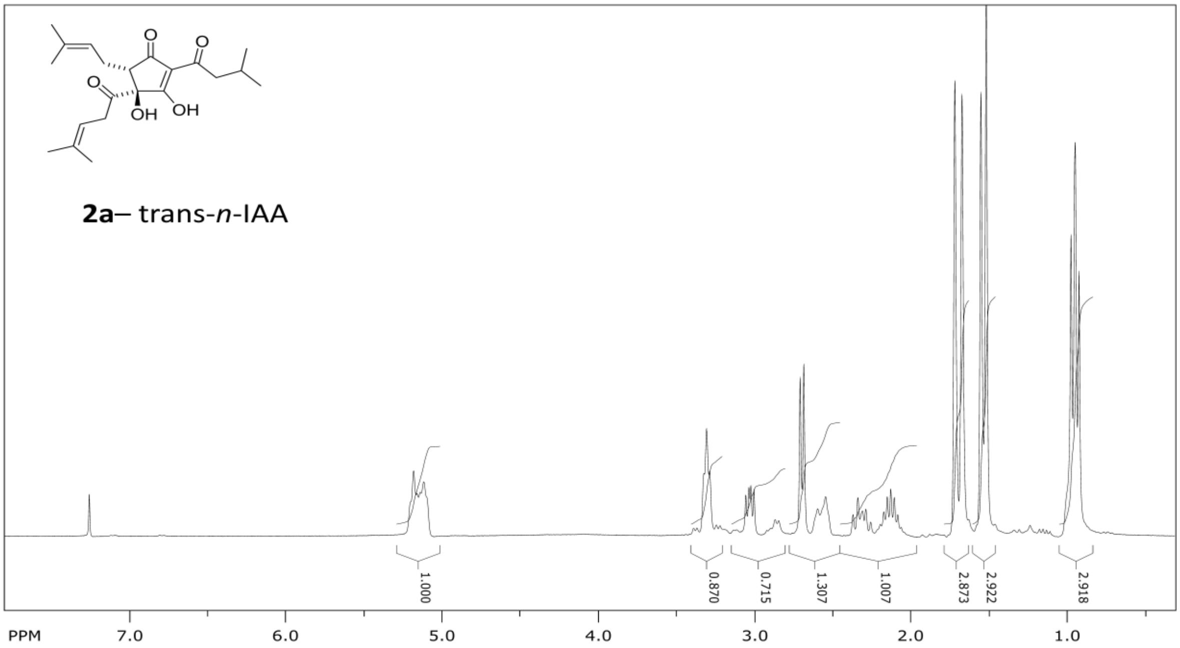

file: E:\BCH3-105-4\10\fid expt: <zg30>
transmitter freq.: 300.131853 MHz
time domain size: 65536 points
width: 6172.84 Hz = 20.5671 ppm = 0.094190 Hz/pt
number of scans: 16

freq. of 0 ppm: 300.130015 MHz
processed size: 32768 complex points
LB: 0.300 GF: 0.0000

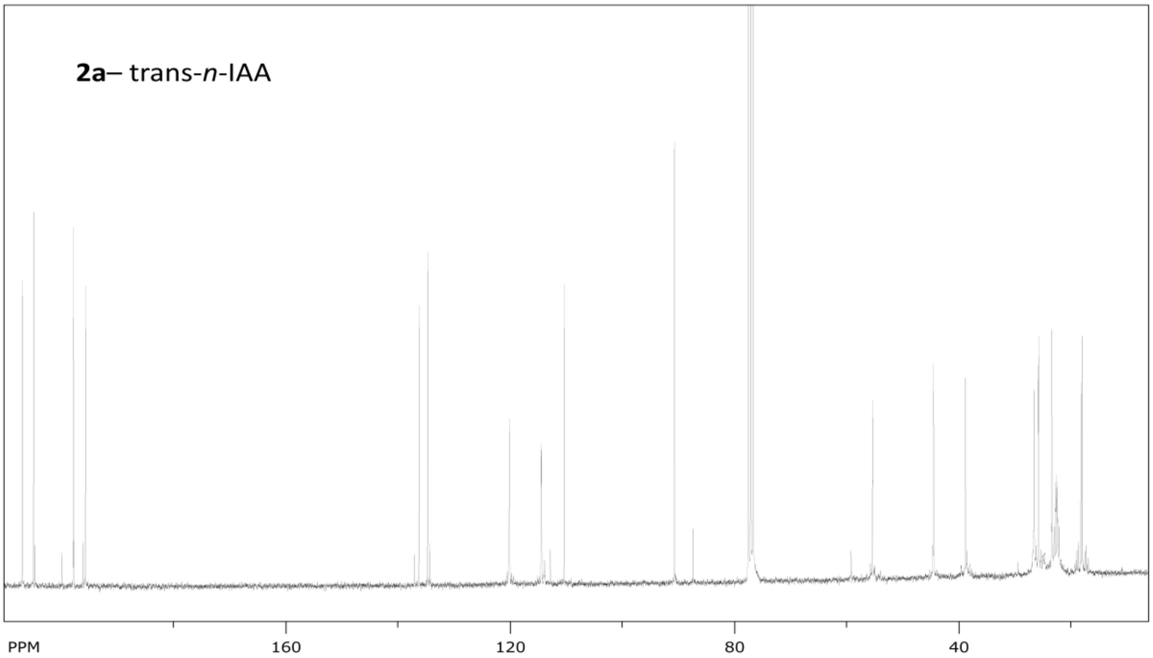

file: E:\BCH3-105-4\RSL\10\fid expt: <zgpg30>
transmitter freq.: 75.475295 MHz
time domain size: 65536 points
width: 17985.61 Hz = 238.2980 ppm = 0.274439 Hz/pt
number of scans: 10240

freq. of 0 ppm: 75.467749 MHz
processed size: 32768 complex points
LB: 1.000 GF: 0.0000

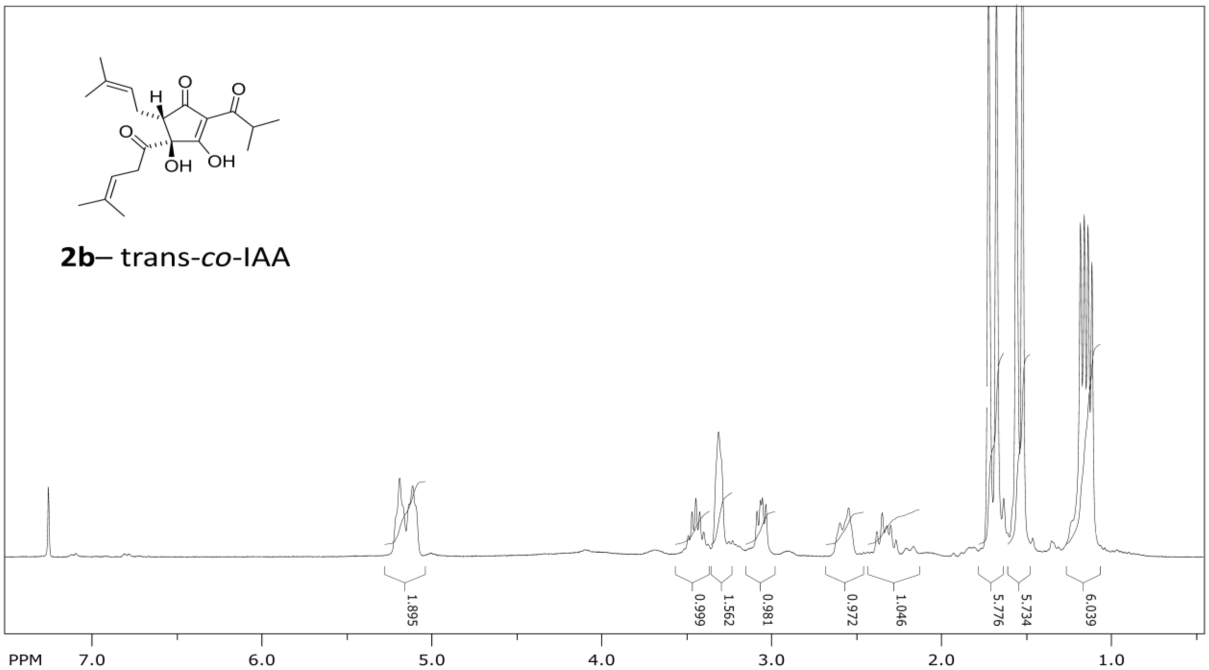

file: E:\bch3-099-2\1\fid expt: <zg30>
transmitter freq.: 300.131853 MHz
time domain size: 65536 points
width: 6172.84 Hz = 20.5671 ppm = 0.094190 Hz/pt
number of scans: 16

freq. of 0 ppm: 300.130015 MHz
processed size: 32768 complex points
LB: 0.300 GF: 0.0000

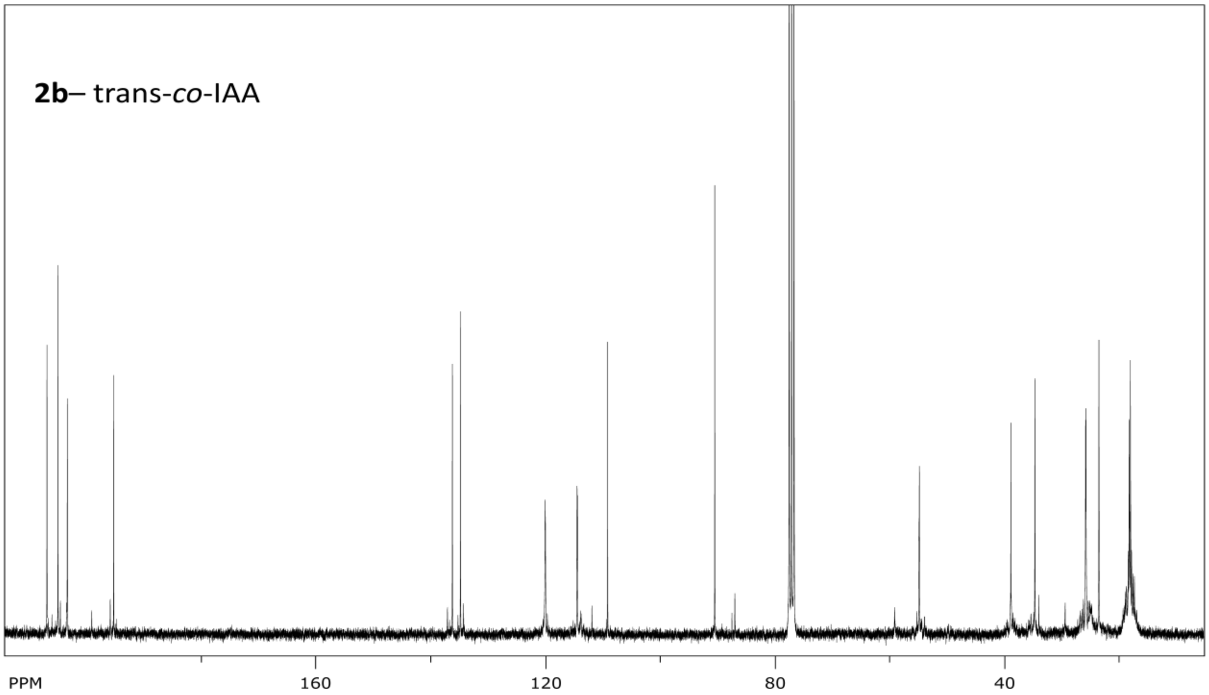

file: E:\BCH3-099-2-H\bch3\10\fid expt: <zgpg30>
transmitter freq.: 75.475295 MHz
time domain size: 65536 points
width: 17985.61 Hz = 238.2980 ppm = 0.274439 Hz/pt
number of scans: 10240

freq. of 0 ppm: 75.467749 MHz
processed size: 32768 complex points
LB: 1.000 GF: 0.0000

7. LCMS Data

General Methods

The LC/MS analysis was performed using an Agilent 1290 Infinity II LC system coupled to an Agilent 6220 TOF mass spectrometer. The instrument was equipped with an electrospray ionization (ESI) interface operating in negative ion mode. The separation was achieved on a ZORBAX column (30 mm × 2.1 mm, 3.5 µm; Agilent Technologies). The mobile phase consisted of water with 0.1% formic acid (solvent A) and acetonitrile with 0.1% formic acid (solvent B). The gradient program was set as follows:

- 0-2 min: 40% B
- 2-8 min: 40-70% B

The flow rate was maintained at 0.4 mL/min, the column temperature was set at Ambient, and the injection volume was 2 µL.

The mass spectrometer was operated under the following conditions:

- Capillary voltage: 3500 V
- Drying gas temperature: 325°C
- Drying gas flow rate: 5 L/min
- Nebulizer pressure: 30 psi
- Fragmentor voltage: 175 V
- Skimmer voltage: 65 V
- OptcopoleRFPeak: 250

Data were acquired in the mass range of 100-1700 m/z.

Data acquisition and analysis were performed using Agilent MassHunter software.

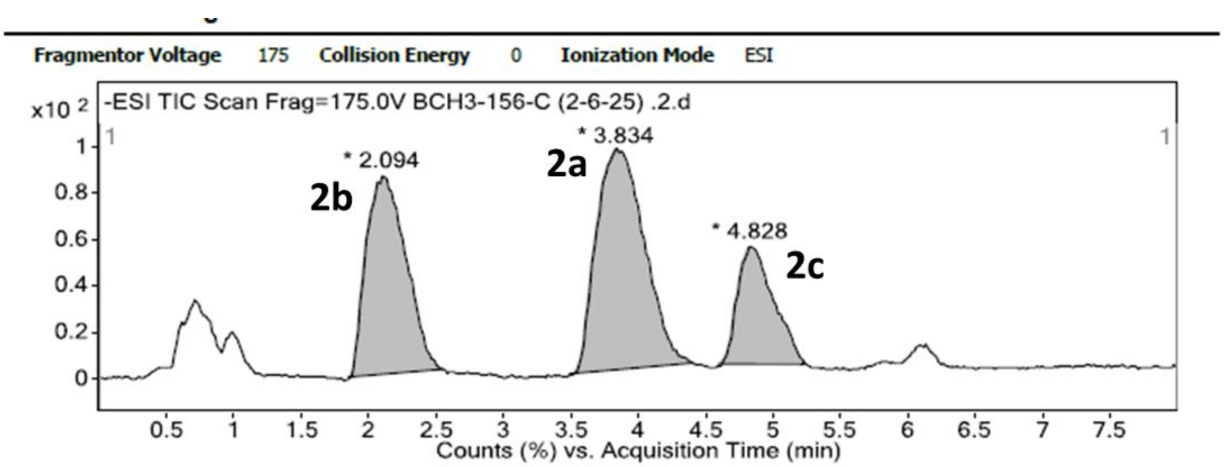

Integration Peak List

Peak	Start	RT	End	Height	Area	Area %
1	1.845	2.094	2.558	11327700.17	233099422.7	74.52
2	3.486	3.834	4.413	12647985.27	312810008	100
3	4.596	4.828	5.225	6721545.05	126271955.1	40.37

Figure S13. Total Ion Current (TIC) chromatogram of mixture of trans-iso-humulones **2a-c.**

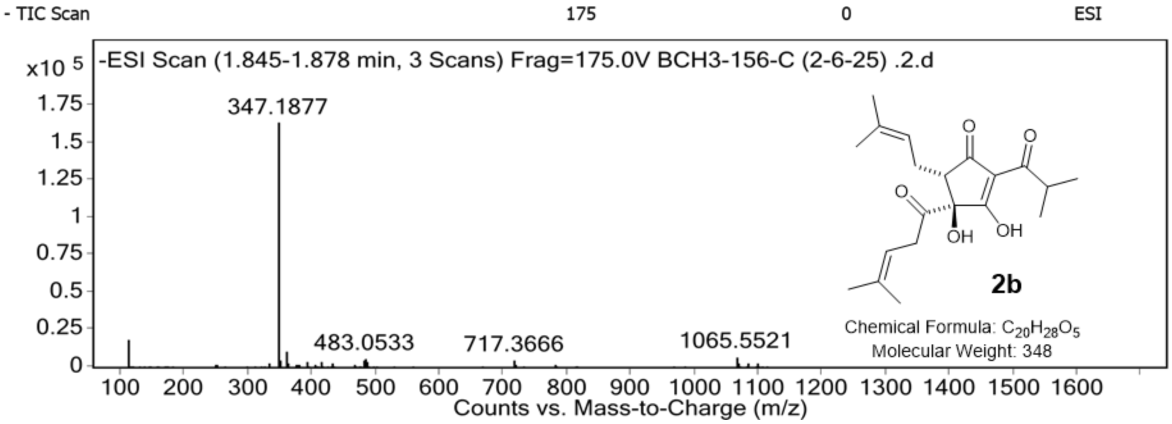

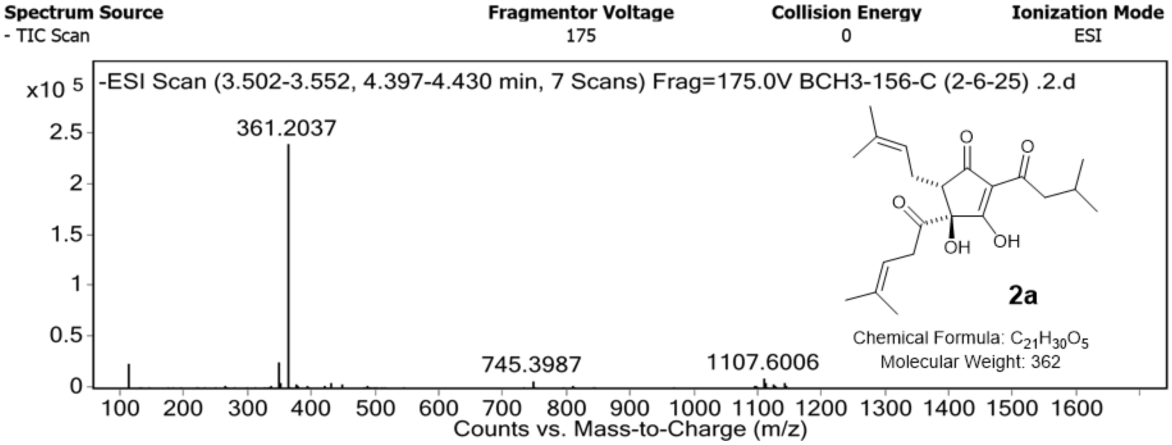

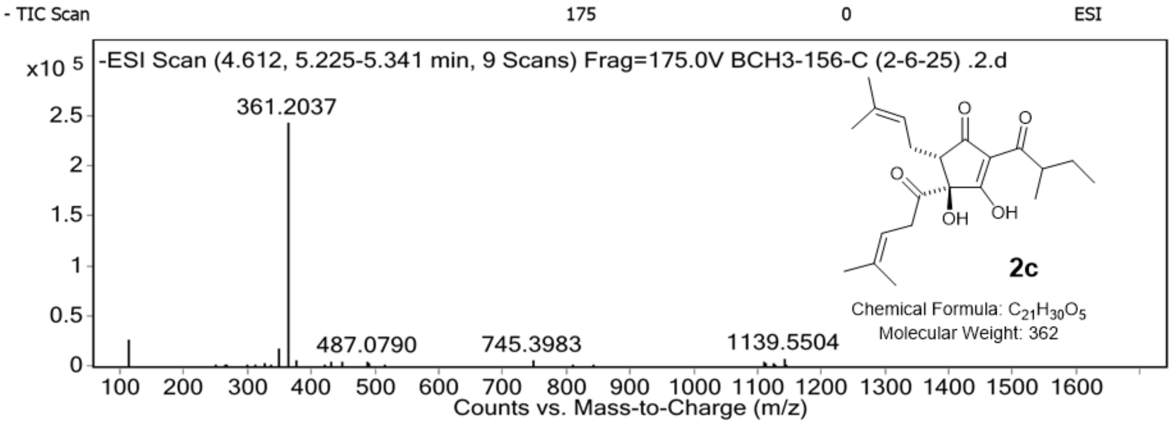

Figure S14. Mass Spectra of trans-iso-humulones **2b**, **2a** and **2c**.
